# Insights into Nanomedicine for Head and Neck Cancer Diagnosis and Treatment

**DOI:** 10.3390/ma15062086

**Published:** 2022-03-11

**Authors:** Cláudia Viegas, Daniela S. M. Pereira, Pedro Fonte

**Affiliations:** 1 Center for Marine Sciences (CCMar), Gambelas Campus, University of Algarve, 8005-139 Faro, Portugal; viegas.claudiasofia@gmail.com; 2 Faculty of Sciences and Technology (FCT), Gambelas Campus, University of Algarve, 8005-139 Faro, Portugal; 3 Faculty of Medicine and Biomedical Sciences (FMCB), Gambelas Campus, University of Algarve, 8005-139 Faro, Portugal; 4 Life and Health Sciences Research Institute (ICVS), School of Medicine, University of Minho, Largo do Paço, 4700-000 Braga, Portugal; daniela.s.pereira30@gmail.com; 5 ICVS/3B’s, PT Government Associate Laboratory, Largo do Paço, 4700-000 Braga, Portugal; 6 IBB—Institute for Bioengineering and Biosciences, Department of Bioengineering, Instituto Superior Técnico, University of Lisbon, 1049-001 Lisboa, Portugal; 7 Associate Laboratory i4HB—Institute for Health and Bioeconomy at Instituto Superior Técnico, Universidade de Lisboa, Av. Rovisco Pais, 1049-001 Lisboa, Portugal

**Keywords:** head and neck cancer, nanomedicine, drug delivery, nanocarrier, hyperthermia, target therapeutic, theragnostic

## Abstract

Head and neck cancers rank sixth among the most common cancers today, and the survival rate has remained virtually unchanged over the past 25 years, due to late diagnosis and ineffective treatments. They have two main risk factors, tobacco and alcohol, and human papillomavirus infection is a secondary risk factor. These cancers affect areas of the body that are fundamental for the five senses. Therefore, it is necessary to treat them effectively and non-invasively as early as possible, in order to do not compromise vital functions, which is not always possible with conventional treatments (chemotherapy or radiotherapy). In this sense, nanomedicine plays a key role in the treatment and diagnosis of head and neck cancers. Nanomedicine involves using nanocarriers to deliver drugs to sites of action and reducing the necessary doses and possible side effects. The main purpose of this review is to give an overview of the applications of nanocarrier systems to the diagnosis and treatment of head and neck cancer. Herein, several types of delivery strategies, radiation enhancement, inside-out hyperthermia, and theragnostic approaches are addressed.

## 1. Introduction

The words cancer, tumor, and neoplasia are generic terms that define a broad range of diseases characterized by unchecked division or uncontrolled proliferation of cells in a tissue or organ. Due to their high rate of division, these cells may invade the adjacent tissues or disseminate at a distance, in a process known as metastasis [1]. This disease can affect any organ or part of the body, and has many histological types. It has a great impact on the quality of life, not only for the patient but also for his family, particularly at a psychological and emotional level, and is often associated with financial hardship [2,3]. Moreover, it is the largest cause of morbidity and mortality worldwide. It is even the leading cause of death in developed countries, and the second in developing countries [3,4]. One of the main factors associated with the increase in the global cancer burden the level of human development, which includes the factors of population growth and aging, changes in lifestyle, economic variables, and social changes [5]. Head and neck cancer (HNC) is a complex and heterogeneous disease that encompasses a large number of cancer cell locations and is considered a leading cause of cancer death worldwide [4,6]. The American Cancer Society refers to HNC as one of the leading causes of death, along with cancers of the digestive system, respiratory system, breast, and reproductive system [7]. Though the various etiological risk factors are well defined, and advances have been made both in its diagnosis and therapy using different approaches, its morbidity has not significantly reduced over the last decades [8].

In this sense, early diagnosis is fundamental to reducing the involvement of other organs and structures, and to improving the prognosis of the disease once the success of the therapy is promoted by early initiation. Conventional cancer treatment protocols include local surgery combined with adjuvant systemic therapies—namely, the administration of cytotoxic drugs and the application of radiation. However, these are invasive methods and are associated with several serious side effects that compromise the patients’ quality of life, such as severe toxicity, mucositis, dysphagia, xerostomia, radiation dermatitis, hematologic toxicity, neurotoxicity, and ototoxicity [6,8]. The fact that these anticancer drugs cannot discriminate normal healthy cells from tumor cells results not only in side effects, but in, low concentrations reaching the target zones, compromising the therapeutic effect. 

Nanomedicine using nanotechnology-based systems emerged as a new cancer management strategy. It involves a wide variety of nanostructures with small particle size distributions capable of crossing biological barriers and interacting with the target molecules of the therapy, among other structures present in the cells, reducing the effects of treatment on healthy cells [9,10].

Thus, nanotechnology-based systems for HNC management have the potential to emerge as alternatives to conventional treatments, as these systems can offer solutions to the problems encountered in conventional treatments: they not only minimize non-specific delivery failures and cell death, and reduce multidrug resistance, but also maximize the efficacy of therapeutic agents. Since they are nanometer-sized systems, they easily penetrate and permeate through the cell membrane, blood capillaries, and biological barriers [11,12,13,14]. These capabilities are very important in the case of HNC, because in some cases, to reach the therapeutic target it is necessary cross biological barriers such as the blood–brain barrier, which we know reduces drug delivery to the brain [15]. This technology also can help clinicians in the monitoring of the disease, because they allow less invasive, more sensitive, and more specific analysis of the affected structures, and facilitate the diagnosis of the disease at an earlier stage, providing greater chances of total remission and therapeutic success. Moreover, nanocarriers are not only biocompatible, but can deliver various diagnostic probes with the ability to target specific biomolecules. Therefore, they can help to provide important structural and metabolic information about a tumor more successfully than other imaging techniques, and identify hidden metastases. Moreover, they can also function as optical contrast agents during image-guided surgery [16,17]. 

With the advantages of nanomedicine in mind, we created this review on nanomedicine-based strategies for HNC diagnosis and treatment. Thus, the main aim of this work is to explore novel and current nanotechnology-based carriers for HNC management, such as drug delivery systems, radiation enhancement, inside-out hyperthermia, and theragnostics.

## 2. Anatomophysiology of the Head and Neck

The head is the upper part of the body that is attached to the rest of the body through the neck. It is the structure that contains the encephalon, which is responsible for our entire state of consciousness (creativity, ideas, imagination, responses, decisions, and memory), along with its protective coatings, the ears, and the face that ensures our identity [18]. 

**Figure 1:**
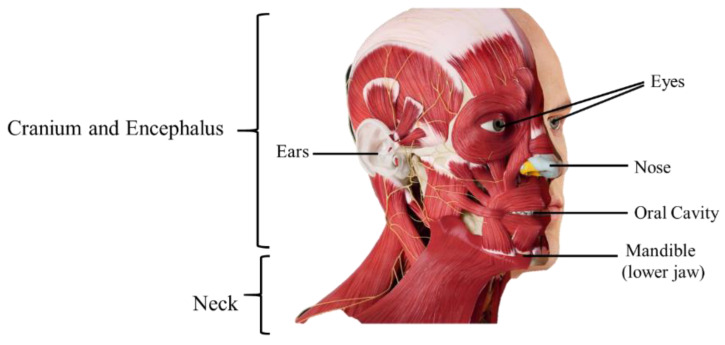
Anatomical structures of head and neck.

In the head (Figure 1), there are also the sensitive receptors (eyes, mouth, ears, and nose); the structures for transmission of voice and expression; and the entrances for nutrients, water, and oxygen and the exit of carbon dioxide. The skull, the bony part of the head, supports the face and protects the brain, and it is composed of two parts, the *neurocranium* (bone box of the encephalon and cranial meninges) and the *viscerocranium* (bones of the face). The *neurocranium* is covered with subcutaneous tissue and skin (part of this skin forms the scalp) [18]. For the face there is a set of bones, the facial bones, which form the nasal cavity, surround the eyeballs, and support the teeth of both jaws. The skull of an adult has 22 individual bones, of which only one is a moving bone, which is the mandible (lower jaw). The remaining 22 bones are immobile and are united in a single unit [19]. 

The neck is the transition zone between the base of the cranium above, and the clavicles and cervical spine below. It connects the head to the torso and limbs, being an important structure between them. It contains numerous vital structures with essential functions for normal physiology, such as breathing, speaking, swallowing, and the regulation of metabolism. In it are several important organs and tissues, such as the larynx, trachea, thyroid, parathyroid, esophagus, and vertebrae [18]. There are also the structures for circulatory and lymphatic inflow and outflow from the head. The main arterial blood flow to the head and neck comes through the carotid arteries, and the main venous drainage occurs via the jugular veins. Lymph from those structures drains into the cervical lymph nodes. The neck has important muscles, such as the platysma, a thin muscle spanning the upper chest to the cheek and lower lip, drawing the lower central lip. It is also the center of a multitude of nervous system structures, transmitting nervous signals from the brain to the body. On the front side of the neck is the thyroid cartilage, the largest cartilage around the larynx and trachea. The neck is slim to allow for the flexibility needed to position the head and maximize the efficiency of its sensitive organs (eyes, ears, mouth, and noise). It is therefore a region that is characterized by fragility and vulnerability. In addition, some vital structures, such as the trachea, esophagus, and glandule of the thyroid, have no bone protection [18,20]. 

**Figure 2:**
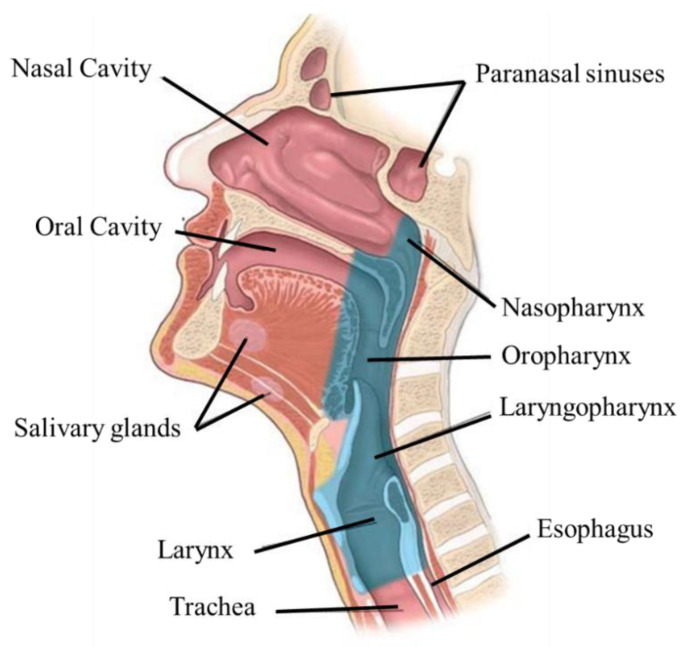
Structures and organs of the upper aerodigestive tract.

The upper aerodigestive tract is located throughout the head and neck region. It includes the oral cavity, oropharynx, laryngopharynx, larynx, nasopharynx, salivary glands, and nasal cavity (Figure 2). The production and articulation of speech, swallowing, and respiration are functions controlled by the oral cavity, pharynx, and larynx. In addition, these structures also protect the airways [21,22]. Damage to one of these structures may affect the functioning of the others. For example, during swallowing it is essential to protect the airways; otherwise, aspiration may result. The oral cavity, besides being involved in phonation, also controls the voluntary phase of swallowing, namely, chewing, preparing the cake, and delivering it to the oropharynx [22].

## 3. Head and Neck Cancer

HNC covers the set of diseases involving abnormal soft tissue growth located in these anatomical areas: deeper than the skull and above the clavicles, except those located in the eye, brain, and esophagus. In addition, it must be malignant, showing invasion of neighboring tissues and dissemination into the bloodstream. These cancers start in the epithelial layer of the mucosa of the upper aerodigestive tract, and consequently include malignant neoplasms originating in the oral cavity, nasopharynx, oropharynx, laryngopharynx, larynx, paranasal sinuses, and salivary glands [22,23,24]—for instance, sarcoma, lymphoma, and salivary gland carcinomas [21]. The pharyngeal area is one of the most common sites affected among the different parts of the oral cavity [13].

### 3.1. Epidemiology, Etiology, and Risk Factors

According to data from 2021, every year, about 932,000 new HNC cases are registered and there are about 467,000 HNC deaths [4]. Neoplasms originating in this anatomical area are some of the most common cancers worldwide, as HNC cases represent about 6% of all cases of cancer [23,24,25,26,27]. The incidence of HNC varies depending on geographic region, population, gender (more common in men), and exposure to diverse risk factors. The major risk factors include tobacco, alcohol consumption, and human papillomavirus (HPV) infection, these being recognized as the main causes of upper aerodigestive cancers in industrialized regions [11,13,24,26,28,29,30]. However, HNC results from several factors, including genetic predisposition, environmental exposure, and behavioral/lifestyle factors. The use of tobacco and alcohol is responsible for about 72% of all HNCs, 4% being due to the use of alcohol alone, 33% to tobacco alone, and 35% to the combined use of these. Thus, the cooccurrence of smoking and alcohol consumption increases the chance of developing HNC, though smoking is considered to be a more major risk factor than alcohol for this type of cancer [29,31]. That said, alcohol is a trigger for the tobacco promoter effect in neoplasm formation [21]. After smoking cessation, there is a reduction in relative risk; however, an individual who was a heavy smoker has triple the risk of a non-smoker, even after 10 years of cessation [21].

Some occupational and environmental contexts have been related to increased incidence of HNC, such as agricultural activities and working a as a cook, waiter, firefighter, butcher or meat preparer, knitter, or roofer. These associations exist because these work environments and occupations are more conducive to smoking and/or alcohol consumption [30]. HPV infection, particularly subtype 16, and to a slighter extent, subtype 18, is believed to be a risk factor for oropharyngeal cancer, based on results in a young non-smoking population [26,29]. Patients with oropharyngeal cancer initiated by the virus typically showed better therapeutic results, and consequently a higher overall survival rate; thus, it is possible to note that age may function as a protective factor, being the reason for the increased survival of this group. Additionally, the augmented expression of p16 protein in HPV-related tumors has significantly better disease-specific survival when compared with non-virus-related tumors that do not exhibit increased p16 protein expression [29]. Infection with this virus promotes an uncontrolled cell cycle which results in genetic instability which, over time, promotes the transformation of premalignant lesions into invasive squamous cell carcinomas. In the case of oropharyngeal squamous cell carcinoma, the stage of development of the virus is an independent prognostic factor for overall survival and progression-free survival [32,33].

Epidemiological studies show that, although the previously-mentioned risk factors are the main ones for most HNCs, nasopharyngeal cancers usually present a set of common etiological factors that include, in addition to those described above, Epstein–Barr virus (EBV) infection and processed food [24,34]. EBV is a DNA lymphotropic herpesvirus that is responsible for the presence of infectious mononucleosis and is highly prevalent in healthy individuals, affecting more than 90% of individuals worldwide [35]. This virus is not found in tumor cells exclusively; however, it is not present in normal cells of the nasopharyngeal epithelium, which implies a direct relationship between EBV activation and the pathogenesis of the tumor [34]. Regarding the co-presence of the above-mentioned viral infections and HNC, a study performed by Al-Thawadi et al. showed that HPV and EBV oncoviruses are co-present in squamous cell carcinomas, especially when they occur in the oral cavity, which may promote their initiation and/or progression; however, the mechanisms of this relationship need to be better elucidated [36].

### 3.2. Pathophysiology

Understanding the origin and pathophysiology of the HCN is fundamental to predicting and managing the course of the disease, and its impact on the patient’s quality of life. This process facilitates the choice of the most appropriate treatment or combination (surgery, radiation therapy, and chemotherapy) while also minimizing possible sequels, such as significant acute and chronic damage to the oral cavity, which is not limited to the hard tissue (teeth and alveolar bone) and the oral mucous membrane, but also affects the soft tissues of the head and neck [37]. Generally, all these malignancies are epithelial because they develop on the upper layers of the epidermis (mucosa) of the upper aerodigestive tract, squamous cell carcinoma being the most common histological type of head and neck tumor. These tumors can range from poorly to well differentiated, and in fact, about 90% of all HNC are squamous cell carcinomas and variants [23,28,38].

In the presence of premature lesions, leucoplakia and erythroplakia with histologic features of hyperplasia or dysplasia are evident. Both cases may deform into invasive tumors, but erythroplakia presents a higher risk of transformation [39]. As in other types of cancer, malignant cells also escape recognition and destruction by immune agents and inhibit or manipulate antitumor immune defenses. It is therefore common for patients with these types of cancer to have low concentrations of CD3+, CD4+, and CD8+ T cells, which may persist even several years after curative surgery. The main mechanisms of immune escape used by tumors to grow and target immune cells are immune destruction escape, tumor suppressor escape and cell regulation, reduction of T lymphocyte activity, immunosuppressive cells, and cytokines that control local and systemic effects [28].

Since the affected areas in these types of cancers are adjacent to the respiratory and digestive systems, the same agents that promote the development of cancer cells in HNCs also affect other organs throughout the body, including the lungs. Thus, these tumors may not appear in isolation, but rather in association with other secondary tumors [40]. As in other types of cancer, angiogenesis is also a determining factor in the development of neoplasm and progression of tumors and is regulated by several endogenous proangiogenic and antiangiogenic factors. Fundamental factors for the growth of cancer and metastasis are the vascular endothelial growth factor (VEGF) and its receptors. This receptor can be upregulated and has significant importance in the prognosis of several HNCs [22,28]. Another important marker that has high expression in HNC is the epidermal growth factor receptor (EGFR), which is expressed in more than 90% of tumors. EGFR is highly expressed in normal epithelial cells, so alterations in its pathways can promote a malignant transformation of HNC [32]. Their expression levels correlate with worsened disease-free survival and overall survival [22,32]. 

HNC can also result from mutations in various genes and pathways, including both tumor-suppressor genes and oncogenes. Some biomarkers aid in the screening, diagnosis, and management of the disease. TP53 and CDKN2A/P16 are mutated tumor-suppressor genes frequently observed in HNC that may confer growth advantages to cells and encourage the development of carcinoma. FAT1 is one of the latest genes implicated in HNC that participates in cell cycle regulation and proliferation, and it is described as a tumor suppressor gene. NOTCH1 is the most recent cancer gene associated with HNC development. Functionally, the gene signaling has both oncogenic and tumor-suppressive roles depending on the cellular context; however, its exact role in pathogenesis needs to be better elucidated. The RAS gene family involves three oncogenes whose mutations in their cell cascades are included in approximately one-quarter of all human cancers. The PIK3CA pathway is another critical pathway in HNC carcinogenesis [32]. Another marker that is sometimes overexpressed in HNC is programmed death-ligand 1 (PD-L1), a transmembrane protein that acts as a co-inhibitory factor of the immune response, reducing the patient’s immune response to tumor cells. Its presence is thus associated with a negative prognosis [41,42]. Advancements in the knowledge of these molecular structures (receptors) and genetic changes which are biomarkers for HNC are important, as they can be potential targets for therapy and help to define new diagnostic and therapeutic strategies, namely, those concerning personalized therapeutics.

### 3.3. Signs and Symptoms

The first symptoms that may indicate the presence or prognosis of HNC are complaints about the aerodigestive tract that are not resolved the conventional treatments. Usually, the tumors in the oral cavity initially start with lesions in the gingivobuccal sulcus tongue, buccal mucosa, or floor of the mouth. Some patients also present with a nonhealing ulcer, an area of irritation between adjacent teeth, pain, or bleeding that does not cease within a normal timeframe. Some other HNCs may be asymptomatic until they are advanced or have developed metastases. 

In the presence of the suspicious signs and symptoms above, it is essential to perform a biopsy for a definitive diagnosis. Another sign that the development of tumor tissue can trigger is the presence of paranesthesia in the oral or nasal cavity because of nerve compression, one of the most common presentations being numbness of the lips and tongue. Lesions in the alveolar ridge can cause loss of malocclusion teeth, or poorly fitting dentures, and tongue lesions can make it difficult to chew. If these symptoms are found in patients with a history of tobacco or alcohol use, the tumor may already be advanced [22].

The lesions in the oropharynx have more insidious symptoms, which can be confused with other diseases or problems, since they can cause pain during swallowing or otalgia. Tumors in the pharynx or supraglottis can cause neck masses because of metastatic lymphadenopathy, though these can be too small to cause other symptoms and be easily detected by a physical examination. Another symptom of this type of cancer is weight loss, not only because of one’s difficulty in swallowing or dysphagia, but also because of the effects on the metabolism. Tumors in the laryngopharynx in advanced stages can reach the larynx, resulting in hoarseness or vocal cord paralysis. The tumors in the glottis are usually the ones that are easiest to access, thereby allowing diagnosis in earlier phases. Early diagnosis is also helped by the vocal cord infections and hoarseness which can occur. In advanced stages, these tumors can compromise breathing [22]. Thus, identifying recurrent symptoms at an early stage is essential for successful treatment.

### 3.4. Diagnosis and Treatment

For the diagnosis of a patient with upper aerodigestive tract complaints, it is essential to understand which areas of the head and neck may cause the symptoms. This procedure is fundamental because the extent of the tumor influences the prognosis and treatment [22,33]. An accurate and early diagnosis is one of the main strategies for successful management of HNC; however, most head and neck tumors are locally advanced at the time of diagnosis, even though they can be easily detected by simple physical examinations [11,22,24]. An evaluation by a multidisciplinary team of the patients medical history, lifelong tobacco and alcohol consumption habits, and the past existence of other cancers and their treatments, including radiotherapy should be questioned [21,33].

The discovery and control of HNC is not always an easy task, since often the affected structures are not accessible to objective clinical examination, resulting in late presentation of the disease. Thus, if any symptom is suspected, an objective inspection of all the structures that may be involved should be performed, including examination of the oral cavity and oropharynx, palpation of the neck, and examination of suspect areas in the mouth. These patients should also undergo transnasal fiber optic endoscopy to examine the pharynx, larynx, and vocal cord structure [21]. Diagnosis will allow evaluating the prognosis of the patient, since it helps to establish the TNM (tumor, node, metastasis) profile of the tumor. TNM staging is commonly used to assess the stages of tumors of the head and neck (although variations are depending on the site of the primary tumor) (Table 1). The “T” classifies the extent of the primary tumor, the “N” refers to the infected regional lymph nodes (it is important to note that the lymphatic drainage of each head and neck subsite is different, so this must be assessed according to the location of the primary lesion), and the “M” refers to distant metastases. With this analysis, it is also possible to establish the stage of the disease, which can vary from 0 to IV, the latter being the stage with the worst prognosis [11,43].

In recent years there has been great progress in defining the staging of head and neck tumors and therapeutic strategies. However, despite all the advances made in the various treatment modalities, the survival rate has remained almost unchanged in the last 25 years. Additionally, since head and neck anatomy are extremely complex, both being composed of several interconnected and interdependent structures, and since typically HNC occurs near structures that are important both at functional and cosmetic levels, early diagnosis is imperative [43].

Despite recent advances in diagnosis, approximately 70% of patients with head and neck squamous cell carcinoma (HNSCC) present with advanced-stage disease, frequently involving regional lymph nodes at the time of diagnosis, leading to high associated mortality. The 5-year survival rate is about 60% [26,28]. Late diagnosis usually implies that the cancer has infiltrated surrounding tissues and spread to the regional nodes, this sometimes being the only clinical manifestation. Furthermore, the invasion of surrounding structures allows the entrance of cancer cells into the bloodstream, which may enable the appearance of distant metastasis and secondary sites; however, distant metastases are not usually present (only in about 10% of patients) [11,24,28,44]. One of the biggest problems associated with this disease is its rate of recurrence. Approximately 50% to 60% of patients with localized HNSCC have their disease progress within two years after diagnosis, which drastically decreases the survival rate (from 80% to 50–35%—depending on the degree of disease progression). Patients with recurrent or metastatic disease have an estimated survival of less than one year [24,26].

Late diagnosis requires aggressive treatments with high morbidity. Most of the time, the treatment will compromise the organs necessary to perform simple functions, such as eating, breathing, and speaking [24,44]. Given the vital importance of the structures involved in these tumors, the therapeutic strategies adopted should not only aim to improve the survival rate, but also preserve the functions of the organs, indicating the need for a multidisciplinary approach [26,28]. The standard treatment for HNC involves surgical resection and radiotherapy (in combination or as isolated treatments) in early stages, and chemotherapy is used in advanced stages of the disease [26,38]. Concurrent chemo-radiation allows preservation of organ function, and it is the main treatment for tumors arising in the oropharynx, nasopharynx, laryngopharynx, and larynx. For oral cavity cancers, the highest cure rates are achieved by using surgical techniques with adjuvant or post-operative radiotherapy (associated or not with chemotherapy) [25]. Radiation therapy is also important in the control and palliation of symptoms in patients with advanced/incurable HNC, allowing tumor reduction, prevention of ulceration and bleeding, and pain control [45]. However, due to the complex anatomy of this region, the conventional approach is always limited, as the treatments can result in severe functional impairment. Surgical resection is usually inadequate due to anatomical limitations, so despite various attempts to improve the existing treatments, they still have severe side effects. The traditional surgical approach is always the preferred treatment, as it removes all macroscopic tumors, yet there is always the concern of having a microscopic disease. With this approach, it is common that malignant cells persist in the tissue margins adjacent to the surgery, meaning that microscopic disease is normally present in the margins of the surgical area, which is often associated with local recurrence and a poor prognosis. Thus, in most cases, radiotherapy is used as well. In addition, surgery can cause severe side effects, resulting in the loss of basic functions and the need for tracheostomy and/or gastrostomy. If a tumor invades the carotid artery or pre-vertebral tissues, it cannot be removed [21,38]. Therefore, if possible, radiation therapy is ideal, due to the reduction in the associated morbidity [38]. This type of treatment can be applied both to the primary tumor and to the lymphatic nodes, and can be referred to as organ-preserving therapy, with or without chemotherapy. However, although radiotherapy is a non-invasive treatment, it is not innocuous, as it can lead to both acute and chronic toxicity in normal tissues. For example, radiotherapy to the head and neck region may cause undesirable radiotherapy-induced changes in the surrounding tissues; and side effects such as oral mucositis, hyposalivation, loss of taste, dental caries, dysphagia, dermatitis (acute) osteoradionecrosis, vessels stenosis, hypothyroidism, hearing loss (late), and trismus, thereby negatively impacting the patient’s quality of life [25,38]. 

Regarding chemotherapy, this treatment modality is not usually employed as an isolated treatment for HNC, but it can still be used in some scenarios and for different purposes [11,38]:

- Radiation enhancement/synchronous chemotherapy, used in conjunction with radiation therapy—reduces the risk of lymph node metastasis;
- Neo-adjuvant/induction chemotherapy—to reduce the tumor’s size before the main treatment;
- Adjuvant—acts on the small lesions that cannot be removed by surgery, reduces the recurrence rate, and improves the survival rate;
- Palliation—for distant metastases.

Currently, the main chemotherapy drugs used in the treatment of HNC are antimetabolites, platinum compounds, taxanes like fluorouracil (5-FU), methotrexate (MTX), cisplatin, carboplatin, docetaxel, and paclitaxel; and various therapeutic protocols can be used, depending on the evolution and stage of the disease [11,46]. One of the standard combinations used to treat recurrent/metastatic HNC non-expressing PD-L1 is cisplatin/5-FU/cetuximab, which allows an increase in the speed of response to therapy, although toxicity may also be higher than the alternatives [47,48]. In the case of chemotherapy induction, the standard regime is cisplatin (100 mg/m^2^) on days 1, 22, and 43 of concomitant radiotherapy [48]. The combination of docetaxel (75 mg/m^2^) and low doses of cisplatin (75 mg/m^2^) and 5-fluorouracil (750 mg/m^2^) each day, for five consecutive days, is also used. This strategy has been shown to reduce the progression of distant metastases, particularly in high-risk patients [49]. In addition to these classes of compounds, EGFR inhibitors have also emerged as a new treatment strategy for HNC. Another method is using certain antibodies that can recognize receptors in cancer cell membranes, leading targeted cell death. Cetuximab (2006) was the first monoclonal antibody to be approved, and demonstrated considerably improved overall survival in patients with locally advanced and recurrent or metastatic HNC tumors. It also showed the role EGFR signaling pathways play in the treatment of HNC [28,32,50]. Nivolumab (2016) was the second antibody approved by the Food and Drug Administration (FDA) for cases of metastatic or recurrent HNSCC. More recently, in 2019, came the approval of pembrolizumab as a first-line treatment for patients with unresectable metastatic or recurrent HNSCC [50]. 

When used in combination with radiotherapy, drugs have more severe and longer lasting side effects than when used alone. This strategy is used for advanced tumors and is reported to be superior to surgery or radiotherapy alone by 6–8% in terms of 5 year survival [21,25,51]. Despite the advances made recently, focused on advanced treatments to preserve organ function and improve quality of life, most of these treatments for HNC have low efficacy, detrimental side effects, and associated morbidities, such as systemic toxicity and cosmetic damage due to lack of selectivity of the therapeutic agents and the invasiveness of the surgical procedures [26,52]. Most of the chemotherapy drugs lack specificity to tumor cells. Thus, they have negative effects on healthy cells as well, resulting in severe side effects, and usually, the concentration of drug achievable at the target is limited, resulting in suboptimal treatment [11,52]. In this sense, the patients subjected to these therapies require significant support from a multidisciplinary team for psychological and physical rehabilitation, including a speech and language therapist, a dietician, a restorative dentist, and a hygienist. However, after treatment, some side effects can persist, despite intensive rehabilitation. A small number of patients never return to a normal oral diet [21]. For that reason, research has been focusing on targeted cancer therapies to prevent these effects. 

The progress in immune checkpoint inhibitors (ICIs) for HNC came to change the therapeutic landscape of the disease, and led to a remarkable benefit for some patients. These are a class of drugs that bind to proteins present in cell membranes that are produced by immune cells such as T cells and some cancer cells, having the ability to block them. These proteins function as checkpoints, allowing the differentiation of self from non-self antigens, and when blocked, can facilitate signaling and mobilization for cell death by circulating T-lymphocytes. The main checkpoint proteins found in cancer cells involved in this type of response in HNC are PD-L1 and CTLA-4 [50]. This therapeutic strategy in combination with other conventional ones, can generate long-lasting immune responses and may lead to a significant improvement in therapeutic efficacy and survival in patients with advanced HNC. An example of this therapeutic approach is pembrolizumab, an anti-PD-L1 antibody that has been approved as a first-line treatment for patients with recurrent or metastatic HNC. It was beneficial in some cases; however, only 20% of patients with advanced HNC who received it showed effective responses [53]. Additionally, the efficacy of these monoclonal antibody therapies is higher when the patients have PD-L1-expressing tumors, making this therapeutic quite specific [48]. Additionally, the majority of patients present primary resistance to ICIs, have several immune-related adverse events, and do not benefit from the use of these agents, emphasizing the need for developing predictive biomarkers to better determine who will benefit from treatment with ICIs and to reduce severe systemic toxicity [53,54].

Moreover, like conventional cytotoxic therapy, immunotherapy is hampered by transport problems. Additionally, this difficulty of membrane permeation into solid tumor tissue by an ICI compromises the efficacy of such therapy. Therefore, it is urgently necessary to optimize the transport strategy of these therapeutic agents, namely, by using nanotechnology, as it can allow the transport of drugs selectively into tumor tissue, minimize toxicity in healthy tissues, and reduce immune-related side events. 

Overall, given the drawbacks of these conventional treatments, the need has arisen to develop strategies and innovative technologies that improve the efficacy and safety of HNC therapies while reducing adverse effects and resistances.

## 4. Nanomedicine as a Therapeutic Approach for HNC

**Figure 3:**
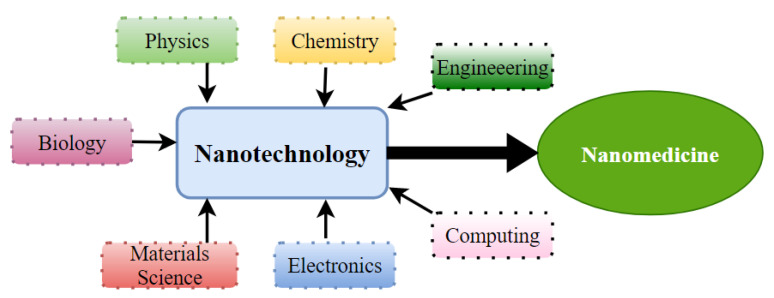
Scientific areas contributing to the use of nanotechnology to nanomedicine.

Nanotechnology is a multifaceted science that combines research fields (Figure 3). Its uses in healthcare come under nanomedicine, which uses molecular tools for diagnostic procedures, treatment, and prevention of diseases (e.g., cancer) [55,56]. Like nanotechnology, the definition of nanomedicine is not agreed on. It concerns a range of particles, which should be at the nanoscale, between 1 and 1000 nm [56,57,58,59,60]. The goals of nanomedicine are common to those of traditional medicine, being earlier diagnosis, the development of non-invasive and effective treatments, and the minimization of side effects [57].

Nanomedicine offers not only improvements for existing techniques, but also the possibility of developing new techniques with superior efficacy, by manipulating drugs at the molecular level and altering their physicochemical properties, such as their solubility and permeability; or facilitating their sustained or controlled delivery [56,57]. Nanotechnology can be applied in numerous fields of medicine, such as imaging [61], drug delivery [62], DNA sequencing [63], and tissue engineering [64]. The arising of applications of nanotechnology in medicine offers new chances for dealing with the problems associated with commonly used therapies. Regardless of its varied applications in medicine, nanotechnology has played a particularly key role in the delivery of drugs using nanocarriers. With cancer being one of the most common diseases worldwide, the treatment of cancer has been the purpose of several studies in the field of nanomedicine. Most have aimed to improve of the existing treatments and at the same time reduce the associated side effects. This type of nanotechnology-based approach improves the overall efficacy and safety of drugs and increases the effectiveness of therapy, as it will condition their variable factors, such as pharmacokinetics, toxicity, targeted delivery, and stability [65].

The Nanomedicine Strategic Research and Innovation Agenda (2016–2030) from the European Nanomedicine Community points out the current needs for cancer treatment [65]:

- The improvement of diagnosis and the development of novel strategies for early detection of tumors, circulating tumor cells, and metastases;
- The improvement of treatment of solid tumors and chemo-resistant tumors;
- The improved precision and efficacy of radiotherapy, immunotherapy, photodynamic, individualized, and hyperthermia therapies
- Lower side effects through more targeted chemotherapy.

Therefore, nanomedicine enables us to address these challenges and improve cancer treatment. One of the main reasons for conventional HNC treatments’ failures is the inefficiency of the doses of therapeutic agents, as little of most drugs effectively reaches the tumor, partly caused by the dose-limiting profile of healthy tissues. The controlled delivery of drugs has become increasingly important because of the advantages that this can bring to the pharmaceutical industry and cancer treatment: it allows a higher drug concentration to reach the tumor cells, while reducing the dose of the drug being administered [26,66]. Drug delivery to a specific location has emerged as an approach to overcome the disadvantages of conventional treatments of HNC, including the absence of specificity of conventional cytotoxic agents [66,67,68,69]. The development of nanocarriers should consider several particulars—namely, influence the properties of the drug, such as its solubility, release profile, bioavailability, and immunogenicity. In addition, it allows overcoming barriers, such as phagocytic mononuclear opsonization. Additionally, four factors are taken into account for the effective design of nanocarriers, namely, the retention of drug-loaded nanoparticles inside of the human body, the escape from the immune system, the ability to reach the target site, and the effective release of the drug at the target site [13]. 

In addition, with drug delivery, resistance to drugs is avoided [70]. Nanocarriers are a class of versatile materials that can function as drug carriers, diagnostic agents, or targeting ligands for HNC. These carriers can improve the properties of drugs and their pharmacokinetics, depending on the intended use [66,68]. In addition, the use of nanocarriers for controlled delivery allows, due to their reduced size, overcoming certain biological barriers, such as the blood–brain barrier; however, they must fulfill some requirements to be used in HNC [66]. 

Firstly, the size and size distribution are among the most important characteristics of nanocarriers, as they are determinant factors for distribution, toxicity, circulation time, in vivo behavior, and targeting to the HNC [26,52]. The nanocarrier’s size is important because most of the properties of nanomaterials are owed to their small size, which gives them proportionally large surface areas, which is critical for drug delivery purposes [52]. Moreover, larger particles are recognized and cleared by the liver and the reticuloendothelial system [52], though this rapid clearance can be avoided through the use of polyethylene glycol (PEG) [26,52]. The small size can also facilitate cellular uptake; and small particles have large surface areas, exposing the bioactive molecules, leading to faster drug release [26].

Dosage is also an extremely important feature when addressing the targeting of drugs. Drugs used in HNC therapy are administered systemically, which requires high dosages and causes side effects in healthy tissues. Thus, it is necessary to decrease the drug concentration in healthy tissues without compromising the therapeutic dose at the site of interest. This reduction can be achieved by localized release of the drug in the target using controlled drug delivery systems [66,68,69]. With the progress made by studies on tumor biology, genetics, and nanotechnology, medicine has become more personalized. Great variability in therapeutic responses has been shown between patients and between head and neck tumors. In this sense, to personalize the therapeutic approach to be taken with each user, it is essential to understand the biological mechanisms related to the distribution and retention of nanocarriers in tumors [48,71]. 

**Figure 4:**
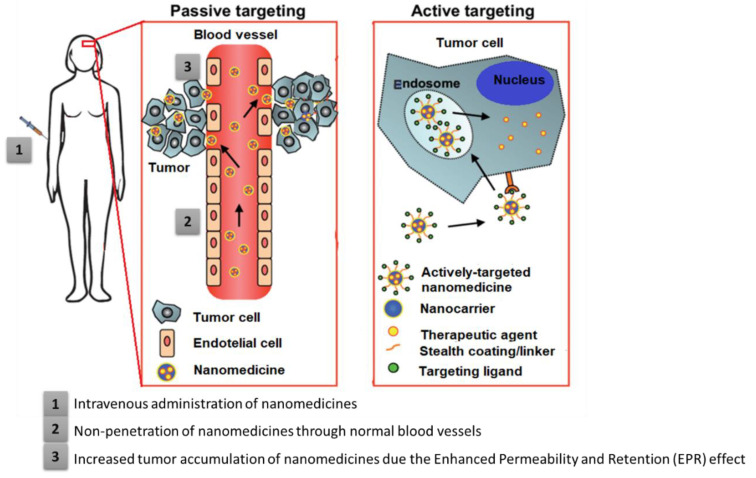
Drug delivery systems’ strategies for anticancer therapy: passive targeting and active targeting. Adapted with permission from [76].

Nanocarriers can be led to the site of interest by passive or active targeting strategies. These strategies aim at increasing the drug concentration in the target cells of HNC, retention, and reducing the toxicity to healthy/systemic tissues [67,68]. While passive targeting takes advantage of the unique characteristics of tumor pathophysiology to drive the nanocarriers to the site of interest without any stimulus or ligand, active targeting makes use of molecules or ligands specific to the site of interest (Figure 4).

### 4.1. Passive Targeting

Passive targeting consists of the systemic injection of the nanocarriers that will accumulate preferentially at the site of interest due to the enhanced permeability and retention (EPR) effect (Figure 4) [11]. It depends on the size of the nanocarriers, and if they are small enough, usually less than 100 nm, their circulation is extravagated through vascular defects typically present at the tumor sites due to accelerated angiogenesis. They should reach hepatic and spleen macrophages [68,70]. This passive targeting effect is also dependent on the degree of vascularization of the tumor, the porosity of the vessels, and the sizes of the pores on the vessels (which vary with the type and stage of the tumor). Solid tumors frequently have leaky vasculature in relation to normal tissues. There is also abnormal lymphatic drainage around these tumors. All these factors promote increased EPR by passive nanocarriers [72,73]. Thus, passive targeting depends on the anatomophysiological conditions of the target. The high production of blood vessels that occurs in tumoral tissues to promote their rapid growth allows carriers such as nanoparticles to be easily retained and accumulated in the tumor tissues [17]. 

The EPR effect can be influenced by a complex set of tumor microenvironment (TME) factors, including tumor characteristics, stage, vasculature, stroma, macrophages, lymphatics, and interstitial fluid pressure, and is therefore difficult to predict [17,74]. Concerning the EPR effect, there are still often some discrepancies between the experiences in animal models used for in vivo assays and human clinical treatments. There are significant size differences between mice and humans, and consequently, the pharmacokinetic profiles and the pharmacodynamic properties of the drugs in the tumors also differ. In this respect, larger animal models may be better benchmarks for estimating the EPR effects of targeted delivery of anti-cancer drugs in humans. Furthermore, in animal models, namely, xenograft mouse models, the effect of EPR may vary between formed and spontaneously implanted tumors. In the case of implanted tumors, the vessels are highly leaky and exaggerate the preferential leakage on nanodrugs. Thus, sometimes good results on solid tumors from animal models can result in disappointing effects when applied to humans, so it is important to take this effect into account when applying a candidate drug to humans [74,75].

### 4.2. Active Targeting

Active targeting consists of the use of a mechanism to increase the specificity of the nanocarrier to the action site [11]. One of the most effective ways to improve the specificity of a nanocarrier is by increasing its affinity for the target by using a molecule with the ability to recognize and bind to the target tissue [77]. Nanoparticles can be given an extra degree of tumor specificity by combining them with targeting ligands. These ligands bind to the nanoparticles’ target during tumor diagnosis/treatment once they interact with the right receptors on the surfaces of the target cells (are tumor-specific markers), and as a result of this interaction, cell internalization occurs [71,76]. All nanoparticles are capable of conjugation with target ligands, such as antibodies/antibody fragments, nucleic acids, sugars, vitamins, peptides, aptamers, and others small molecules. For example, to ensure that adequate contrast is provided during surgery in HNC, the target of interest must be highly expressed while expression is minimal in surrounding healthy tissues. The functionalization of nanoparticles has the advantage of leaving the fluorophore chemically unchanged, thereby limiting the possibility of optical performance [17]. 

The major challenge of active targeting is the choice of targeting agent to avoid toxicity to healthy cells. Another type of active targeting is the so-called physical/triggered targeting, which consists of the use of internal (pH, redox potential, enzymes) or external stimuli (UV light, temperature, ultrasound) to drive the nanocarrier to the site of interest before triggering the release of the drug [78,79]. This targeting is especially notable in the form of magnetic nanocarriers, which can be guided to the site of interest through an external magnetic field.

## 5. Nanocarriers for HNC Diagnosis and Treatment

The usefulness of nanocarriers both as vehicles of diagnostic agents to specific tumor sites and as carriers of therapeutic agents has overcome the disadvantages associated with conventional strategies, improving their efficacy and safety. Thus, in the case of HNC, nanocarriers can be used for drug delivery, as adjuvants in radiotherapy to promote radiation therapy, in local hyperthermia, in plasmonic photothermal therapy, in magnetic fluid hyperthermia, and for simultaneous diagnosis and therapy (theragnostic).

**Figure 5:**
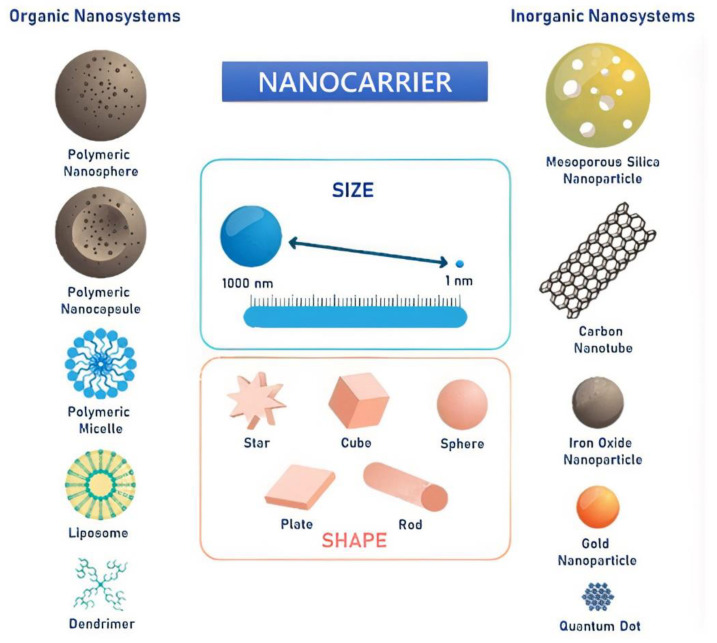
Organic and inorganic nanosystems for drug delivery and diagnostic applications. Size range and different shapes of nanosystems.

There are several types of nanocarriers (Figure 5) that can be applied for HNC diagnosis and treatment (Table 2), such as polymeric nanoparticles, lipid-based nanocarriers, dendrimers, carbon nanostructures, and metallic nanoparticles. They have enormous potential as carriers of bioactive molecules because of their small size and unique physicochemical and biological properties, making them promising for biomedical applications [55,80,81].

Nanoparticles can be divided into two categories, hard and soft (Figure 5). Hard nanoparticles are composed of inorganic materials and include quantum dots (QDs), noble metal, metal oxides, and lanthanide-based nanoparticles. As these nanoparticles are composed of inorganic materials, the potential for toxicity and colloidal stability are significant concerns. The advantage of inorganic nanoparticles is their ability to receive physical and chemical surface modifications. Therefore, they are often surface-modified with biocompatible materials, such as PEG, to increase in vivo safety and performance [17]. 

On the other hand, soft nanoparticles are composed of organic materials and include lipidic carriers, liposomes, dendrimers, and polymer nanoparticles. For surface modifications, soft nanoparticles can be functionalized with a diverse variety of hydrophilic and amphiphilic molecules, such as fluorophores or agents that provide in vivo stability to otherwise poorly soluble molecules or more make them less recognizable to macrophages [17,82,83]. 

Nanoparticles guide drugs to the target cells while avoiding normal cells, increasing the intracellular concentrations of drugs in the target cells and reducing toxicity. They usually can bind to specific receptors on the surfaces of the target cells, and be internalized by endocytosis. With certain materials/structures, this mechanism can avoid recognition by the P-glycoproteins [67,84]. Ideally, nanoparticles used in medical applications should be biocompatible and non-toxic. Other materials used in the production of nanoparticles to provide anti-cancer drugs against HNC, namely, OSCC, are the biocompatible polymer poly (lactic-co-glycolic acid) (PLGA), which has the advantage of being degradable in an aqueous medium; the lactic and glycolic acid; and poly (ethylene glycol)-poly (glutamic acid), which is used as a block copolymer for the delivery of cisplatin [13,85].

The undesirable effects that may arise from the application of nanoparticles depend on the hydrodynamic diameter, shape, chemical groups on the surface, route of administration, and half-life in the bloodstream. In terms of size, small nanoparticles have proportionally larger surface areas, making them more reactive and therefore more toxic [55]. In Table 2. there is an overview of several examples of nanocarriers applied in HNC management which will be described in the next sections.

**Table 2:** Overview of several examples of nanocarriers applied in HNC management.

Nanocarrier	Load	Targeting Moiety	Targeted Cell/Tissue	Application	Ref.
Cationic lipid Nps	pre-microRNA-107	MicroRNA-107	Xenograft model of HNSCC cell lines (SCC15, SCC25, and CAL27 cells)	Targeted pre-miR-107 delivery	[86]
Liposomes	Curcumin-difluorinated	Cancer stem cell marker (CD44)	Cisplatin resistant HNSCC cell lines (CCL-23 and UM-SCC-1, laryngeal and oral cavity carcinomas respectively)	Targeted curcumin-difluorinated delivery	[87]
PEGylated liposomes	^188^Rhenium	*let*-7 miRNA	Hypopharyngeal cancer (FaDu) cells and Human tongue squamous cancer (SAS) cells	Targeted ^188^Rhenium delivery	[88,89]
PEGylated NLC	Cisplatin and paclitaxel	Folate receptor (FR)	FaDu cells	Targeted co-delivery of DDP and PTX	[90]
Liposomes and SLNs modified with a pH-sensitive PEG shell and DSPE-PEG-peptide	Irinotecan and miR-200 microRNA	EMT-associated genes and topoisomerase-I	HNC SAS cells	Targeted Irinotecan and miR-200 delivery	[91]
Phospholipid complex (soybean lecithin, with phosphatidylcholine content of 70–97%) Nps	Salvianolic acid B	-	HNSCC cells lines (HN13, HN30) and Leuk1 precancerous cells	Drug delivery of SalB	[92]
Polymer delivery Nps Atrigel^®^ (leuprolide acetate)	Cisplatin	-	C.B-17 severe combined immunodeficiency spontaneous mutant mouse	Cisplatin delivery	[93]
Polymer micelles -Poly(ethylene glycol)-poly(glutamic acid) block copolymers (PEG-P[Gu])	Cisplatin	-	Oral HNSCC cell lines (OSC-19, OSC-20, HSC-3 and HSC-4)	Cisplatin delivery	[94]
pH-responsive diblock polymers cationic micelles (dimethylaminoethyl methacrylate blocks)	siRNA	Proapoptotic gene (Pkcδ)	Submandibular rat glands	Targeted siRNA delivery	[95]
Biocompatible polymer (poly(e-caprolactone):poly(lactide-cocaprolactone) (PLCL:PCL))	CCL21 and cisplatin	-	HNSCC tumors	Delivery of CCL21 and Cisplatin	[96]
Dendrimers of polyamidoamine (PAMAM)	MTX	FR	HNSCC cell lines (xenografts of no folic acid receptor expression, intermediate folic acid receptor expression and high folic acid receptor expression)	Targeted MTX delivery	[97]
Gadolinium Nps	-	-	Radioresistant HNSCC (SQ20B) cell lines of the larynx after radiation therapy	Improvement of RT	[98]
Gadolinium Nps (AGuIX^®^)	DOTAGA	-	Radioresistant HNSCC cell lines (SQ20B, FaDu, and Cal33—tongue squamous cell carcinoma)	Improvement of irradiation of carbon ions (^13^C^+6^)	[99]
AGuIX^®^	DOTAGA	-	HNSCC cells lines (SQ20B)	Preparation for radiation application	[100]
Silica AuNanoshells	-	-	HNSCC cells lines (Human FaDu cancer cell line—ATCC, HTB-43 and rat alveolar macrophages (NR8383; ATCC# CRL-2192)	Plasmonic photothermal therapy (PPTT) and photodynamic therapy (PDT)	[101]
Gold nanorods (AuNRs)	-	-	HNSCC tumor	Photothermal tumor therapy (PTT)	[102]
Au Nps	anti-EGRF monoclonal antibody (host mouse)	EGFR	Oral squamous carcinoma cells (OSCC)	Targeted drug delivery of anti-EGFR antibody and photothermal agent (laser irradiation)	[103]
Au Nanorods	Rose Bengal	-	OSCC	Photodynamic and photothermal oral cancer therapy	[104]
Dextran-coated superparamagnetic iron oxide nanoparticles functionalized with hyaluronic acid (HA)	-	CD44	Tongue squamous cell carcinoma cells	Magnetic fluid hyperthermia (MFH)	[105]
Superparamagnetic iron oxide nanoparticles	Mouse Anti Human CD44 antibody (sc-7297)	CD44	HNSCC cancer stem cells	MFH	[106]
Magnetic iron oxide Nps	-	-	HNSCC cell line (Tu212) of mouse xenograft model	MFH	[107]
PAA-attached mesoporous Fe_3_O_4_ Nps	Bleomycin	-	Human tongue carcinoma cells (Cal-27) line	Magnetic targeting delivery	[108]
SLN (Compritol ATO888, lecithin, and glycerylmonostearate)	Andrographolide	-	Human immortalized oral epithelial (HIOEC), precancerous leucoplakia (Leuk1), HN6, and HN30 cells	Drug delivery of ADG	[109]
Multifunctional polymer (Linear-dendritic mPEG-BMA4) Nps	Saracatinib	Proto-oncogene tyrosine-protein kinase Src	HNSCC cell lines (HN6—tongue squamous cell carcinoma; HN8 -metastatic lymph node site from oral cavity; HN12—tongue squamous cell carcinoma)	Targeted Saracatinib delivery	[110]

### 5.1. Nanocarriers for Drug Delivery

In this section we focus on the most promising nanocarriers for drug delivery in HNC treatment, the lipid-based, polymer-based, and metallic-based nanocarriers.

#### 5.1.1. Lipid-Based Nanocarriers

Lipid-based nanocarriers are mostly constituted by phospholipids, which, due to their unique characteristics, undergo the process of self-organization when they are in an aqueous medium, forming organized structures. Phospholipids are amphiphilic molecules each having a polar head with a phosphate group—hydrophilic end—and hydrocarbon chains derived from fatty acids—hydrophobic ends. The fact that lipids are amphiphilic molecules allows them to form structures organized by processes called self-assembly. Depending on the nature of the lipid, it may form distinct structures: micelles, lipid bilayers, or liposomes. In drug delivery, the most used lipid-based nanostructures are micelles and liposomes. Micelles are colloidal structures that each contain a hydrophobic center, where the hydrocarbonated tails are located, surrounded by the polar heads. Amphipathic molecules having a polar group and only one hydrocarbon tail are more prone to forming micelles [77,111].

Concerning HNSCC, some studies involving lipid-based systems have been described. After demonstrating that microRNA-107 (miR-107) was significantly downregulated in HNSCC tumors when compared to normal tissues, Piao et al. developed cationic lipid nanoparticles composed of dimethyldioctadecyl ammonium bromide (DDAB), cholesterol, and α-Tocopheryl polyethylene glycol 1000 succinate for pre-miR-107 delivery [86]. The nanoparticle-mediated pre-miR-107 delivery increased the concentration of pre-miR-107 in HNSCC cells, compared to administration of free pre-miR-107. Additionally, survival, cell invasion, and cell migration of tumors cells were repressed by the pre-miR-107 in the presence of nanoparticles.

Liposomes are one of the most studied nanocarriers in the treatment of cancer. They are each composed of an amphiphilic phospholipid bilayer and an inner aqueous cavity, which results in an artificial spherical vesicle that is able to encapsulate molecules with different polarities. Nanocarriers of this type have been used in several formulations already marketed or in clinical trials, to treat several pathologies, especially HNC. Preparation of liposomes was reported for the first time by Prof. A. Bangham in 1965. However, it was Gregory Gregoriadis who established the concept of drug encapsulation in liposomes [112,113]. Since then, liposomes have been described as the ideal nanoencapsulation system because of their ability to carry compounds, overcoming the problems associated with other systems, such as pharmacokinetics, in vivo stability, and toxicity. One example of this is the encapsulation of natural composts, such as curcumin, in liposomes. Curcumin is a polyphenol derived from *Curcuma longa* and is extensively studied due to its potential as an anti-inflammatory, antibiotic, and antioxidant agent, and it also presents anticancer activity [114]. Although curcumin sensitizes HNC cell lines to cisplatin [87], results have not been successful in some human clinical trials, due to poor absorption of curcumin by the gastrointestinal tract. Thus, research has focused on creating curcumin analogues that have better qualities, such as curcumin-difluorinated (CDF), a synthetic analogue of curcumin that is more effective than curcumin in the growth inhibition of several human tumor cell lines. Basak et al. (2015) encapsulated CDF in liposomes and evaluated the growth inhibition in cisplatin-resistant HNSCC cell lines. Growth inhibition in vitro and expression levels of the cancer stem cell marker (CD44), cytokines, and growth factors were investigated after CDF encapsulated liposome treatment, showing significant growth inhibition and a reduction in the expression of CD44, indicating an inhibitory effect of liposomal CDF on cancer stem cells [87]. Another proposed structure for carrying and improving the bioavailability of natural compounds that have been evaluated for HNSCC chemoprevention is that used by Li et al. In their work, they proposed the encapsulation of salvianolic acid B (SalB) in phospholipid complex-loaded nanoparticles (PLC-NPs) as a potential treatment for HNSCC cells (HN13, HN30) and Leuk1 precancerous cells. The results showed that the intracellular accumulation of SalB was higher when HN13, HN30, and Leuk1 cells were in the presence of the SalB–PLC-NPs complex (nano-SalB) compared to cells administered free SalB. Similarly, cell viability was lower and apoptosis was higher in the presence of these nanoparticles compared to free SalB [92].

Liposomes can also function as carriers for other bioactive compounds. Lin et al. evaluated the biodistribution, pharmacokinetics, drug accumulation, and therapeutic efficacy of PEGylated ^188^Rhenium-liposomal nanoparticles (^188^Re-liposome) in HNSCC. ^188^Re-liposome significantly accumulated in the tumor and had a longer circulation time, when compared with the radionuclide alone, showing the enhanced targeting of ^118^Re in encapsulated form. In addition, tumor growth was reduced after a single treatment with ^188^Re-liposome. It was also found that the ^188^Re-liposome up-regulated the tumor-suppressive *let*-7 miRNA [88]. This therapeutic approach was also studied by Chang et al., where different dosages of ^188^Re-liposome were compared for therapeutic efficacy against HNSCC in vivo. Systemic toxicity and markers of tumor proliferation and epithelial-mesenchymal transition (EMT) were analyzed, and dosimetry was used to assess the different dosages of ^188^Re-liposome. A high dosage of ^188^Re-liposome exhibited significant killing effects on hypopharyngeal cancer (FaDu) cells and human tongue squamous cancer (SAS) cells, but not on OSCC (OECM-1) [89]. As in previous studies, they demonstrated high accumulation of ^188^Re-liposome in the tumor, greater with repeated doses when compared to a single dose. Repeated doses also reduced tumor growth rate and extended the survival of tumor-bearing mice. These observations were associated with a significant loss of EMT markers in tumor cells [88]. 

Research in HNC has showed that the combined treatment of cisplatin (DDP) and paclitaxel (PTX) is more effective than a single-drug therapy. However, it is known that these drugs have adverse effects, such as renal toxicity (DDP) and hematological toxicity (PTX). Yang et al. developed PEGylated nanostructured lipid carriers (NLC) functionalized using folate (FA) (FA-DDP/PTX NLCs) for co-delivery of DDP and PTX to HNC cells (FaDu cells). The folate receptor (FR) is highly expressed in cancer cells—namely, in HNC cells—so FA-DDP/PTX NLC enhanced the anticancer efficiency of the drugs, avoiding any obvious toxicity in vivo. These results show that this nanocarrier can be used as a transporter for DDP/PTX co-delivery [90].

A study done by Lo et al. used a combinatory therapy and nanoparticles—chemotherapy and gene therapy co-treatment—as a potential strategy for bypassing therapy resistance, decreasing noncancerous cells’ drug uptake, and improving passive tumor targeting via EPR effect and ligand–receptor binding. They used liposomes and solid lipid nanoparticles (SLN) modified with a pH-sensitive, self-destructive PEG shell, and different peptides were designed as nanovectors for the delivery of irinotecan and miR-200 to enhance tumor-specific accumulation. The miR-200 microRNA regulates EMT-associated genes and has a suppressive effect on them. Irinotecan is a topoisomerase I inhibitor, so it causes cell death by slowing the rewiring of double-stranded DNA. In this work, it was concluded that the cleavable PEG layer was sensitive to low extracellular pH, and the targeting peptides associated with the nanoparticles improved the reuptake and release of miR-200 and irinotecan into HNC SAS cells. This resulted in increased apoptosis of SAS cells treated with this combinatorial therapy, and better therapeutic efficacy and safety, in comparison with the results of the commercially available Onivyde and other formulations in a SAS tumor-bearing mouse model. Thus, this study has shown how combination therapy using pH-sensitive coatings and modified nanoparticle targeting can be a novel strategy for HNC treatment [91]. Another application of SLNs as a vehicle of compounds with anticancer activity in HNC and precancerous cells was in the work performed by Li et al. (2020) for the delivery of andrographolide (ADG) in the human immortalized oral epithelial (HIOEC), precancerous leucoplakia (Leuk1), HN6, and HN30 cells. This study showed that in the presence of encapsulated ADG (ADG-SLN), compared to free ADG, the 50% inhibitive concentrations (IC_50_) against HIOEC, Leuk1, and HN6 and HN30 cells are much lower. Moreover, the results also showed higher efficacy cell cycle arrest and apoptosis, which reveals that the use of SLNs as nanocarriers promotes therapeutic success [109].

#### 5.1.2. Polymer-Based Nanocarriers

Polymer-based nanocarriers are known for their biocompatibility, stability, low toxicity, negligible side effects, and complete degradation inside the human body; and depending on their chemical compositions, polymeric nanoparticles can be produced from synthetic or natural polymers [115]. 

As previously mentioned, most head and neck tumors at the time of diagnosis are already in an advanced stage, presenting alongside metastases in other areas of the body. This is mostly because of the molecules involved in tumor progression, including the proto-oncogene tyrosine-protein kinase Src, a non-receptor tyrosine kinase and one of the most targetable molecules in HNSCC. It is overexpressed and hyperactivated in this type of cancer [116]. Considering this, Lang et al. synthesized multifunctional polymer (Linear-dendritic mPEG-BMA4) nanoparticles for selective release of saracatinib, a kinase inhibitor, into HNC cells and evaluated the anti-tumor efficacy and efficiency of saracatinib-loaded polymeric nanoparticles in vivo. Comparatively, with free drug, the saracatinib-loaded polymeric nanoparticles revealed superior anticancer effects, avoiding metastasis more efficiently [110]. Polymer-based drug delivery was also studied by Chen et al., who reported the transport of cisplatin for the treatment of human HNSCC using an injectable, biodegradable polymer. This biodegradable polymer (Atrigel^®^—leuprolide acetate) released 80% of its cisplatin over 7 days, and had a significantly more powerful tumor suppression effect compared with free cisplatin [93]. This approach was also used in OSCC, by Endo et al. They developed polymeric micelles of poly(ethylene glycol)-poly(glutamic acid) block copolymers (PEG-P[Gu]) carrying cisplatin (NC-6004) and evaluated its effectiveness and safety both in vitro and in vivo. The in vitro growth-inhibitory effect of NC-6004 was significantly lower compared to the free drug; however, NC-6004 showed equivalent antitumor effects in vivo when compared with the free drug. Regarding side effects, the free drug caused renal cell apoptosis, but mice injected with NC-6004 were practically free of renal cell injury [94]. 

Regarding HNSCC treatment and radiation therapy side effects, improved radioprotection strategies have been studied, since radiation may damage the salivary glands (SGs), resulting in hyposalivation or xerostomia. To develop radioprotection strategies for the SGs, targeted delivery of small-interfering RNA (siRNA) for gene silencing therapy was studied by Arany et al. To reach the proapoptotic gene (Pkcδ) before radiation, nanoparticles of pH-responsive composed of diblock polymers forming cationic micelles (dimethylaminoethyl methacrylate blocks), electrostatically bonded to the negatively charged siRNAs, were introduced into rats’ submandibular glands. This treatment has been shown to protect long-term hyposalivation in irradiated animals because after the elimination of Pkcδ, the number of apoptotic cells was reduced, and saliva secretion improved. Thus, it has been demonstrated that the supply of siRNAs carried by nanoparticles was an effective methodology to confer radioprotective protection to SGs. As previously mentioned, it has been demonstrated that the drug combination approach has produced better results in tumor management [95]. Thus, Pellionisz et al. engineered a novel biocompatible polymer loaded with CCL21 cisplatin [96]. The polymer platform is already in use, so this work aimed to compare CCL21-cisplatin loaded in a biocompatible polymer (poly(e-caprolactone):poly(lactide-cocaprolactone) (PLCL:PCL)) and free polymer in terms of decreasing head and neck tumor size. The in vivo experiments showed that tumor volume after the treatment with the drug-loaded polymer was significantly reduced when compared to group treated with the polymer alone, suggesting the antitumor efficacy of polymer and drug combinations, which is made all the better by the reduction in/absence of cytotoxic effects. 

Folate-targeted chemotherapy with methotrexate (MTX) is a common therapeutic approach in recurrent or metastatic HNSCC; however, this type of therapy is often associated with severe side effects [28]. Dendrimer-targeted therapy can decrease systemic toxicity while increasing the efficacy of this drug. To evaluate this hypothesis, Ward et al. identified cell lines with folate receptors and evaluated in vivo the efficacy of G5 polyamidoamine (PAMAM) dendrimer-based targeted chemotherapy on three cell lines with different folate receptor expressions (null, intermediate, and high expression). Additionally, drug efficacy and toxicity were compared between targeted therapy, free MTX, and the saline control. It was found that targeted therapy was effective against high folate receptor expression cell lines and could be delivered in molar doses three times that of free drug, allowing increased efficacy when compared with free MTX and control [97]. 

Similarly to cisplatin, paclitaxel is one of the most used drugs in the treatment of cancer. Thus, several groups have been attempting to perform targeted therapy with paclitaxel [117,118]. Damascelli et al. used paclitaxel carried by nanoparticles made of human albumin as induction therapy before definitive treatment of squamous cell carcinoma of the tongue. These nanoparticles were stabilized by binding albumin (nab) to the hydrophobic drug (paclitaxel)-nab-paclitaxel/albumin nanoparticles. Of the 23 treated patients, 18 had a tumor response, 3 showed stable disease, and only 2 showed disease progression, substantiating the effectiveness of this approach as induction therapy [119]. On the other hand, Haider et al. (2020) showed how optimization of the design of paclitaxel-loaded PLGA (poly(lactic-co-glycolic acid) nanoparticles (PTX-PLGA-nanoparticles) to ultra-small sizes, of about 53 nm and with EE% higher than 90%, allowed a 10-fold increase in drug release in vitro compared to 72 h of the free drug. Furthermore, in pharyngeal carcinoma cells, a 50% decrease in viability was achieved within 24 h after treatment with optimized PTX-PLGA-nanoparticles, whereas this was only 20% with the free drug. These results showed that the optimization of the parameters used in the design of these nanoparticles favored not only their intracellular uptake, but also the efficacy of the antitumor activity of PTX [120]. Another strategy of PTX delivery in polymeric nanocarriers was developed by Riestra-Ayora et al. for the treatment of hypopharynx carcinoma squamous cell (FaDu) tumors xenografted into mouse models. The design of this nanocarrier was based on the combination of block copolymers of polyethylene glycol (PEG) as a hydrophilic domain and a methacrylic derivative of α-tocopheryl succinate (α-TOS) as the hydrophobic domain. The results showed that PTX conveyed in this nanocarrier compared to free PTX had much higher antitumor activity, inducing apoptosis more effectively. It also showed an improved safety profile. These results would have been influenced by the fact that α-TOS can produce high levels of reactive oxygen species (ROS) that activate the apoptosis cascade. Thus, the inclusion of α-TOS in the design of nanoparticles is a promising strategy for HNC therapy, since it potentiates the PTX effect [121].

#### 5.1.3. Metallic-Based Nanoparticles

Metallic-based nanoparticles are also being used as nanocarriers for drug delivery in HNC therapy. One strategy was using superparamagnetic nanoparticles by functionalizing their surfaces. The work was done by Zhang et al., in which they developed a novel drug delivery system based on magnetic nanoparticles for HNCs. This system consisted of a combination of biocompatible mesoporous Fe_3_O_4_ nanoparticles with superparamagnetic properties attached to polyacrylic acid (PAA). The drug used for the treatment of HNC was bleomycin (BLM), which can be either encapsulated within the mesoporous structure of the superparamagnetic nanoparticles or attached to the surface of the PAA polymer via molecular crosslinkers, which works as a polymer shell to reduce the inherent clearance of superparamagnetic nanoparticles while regulating the release of the drug. These nanoparticles with paramagnetic properties delivered BLM to the focal area of the magnetic field in the tumor tissue, allowing for its gradual release and apoptosis of the tumor cells, while reducing the severe side effects of BLM to the normal cells and tissues. This new strategy based on a simple method and without using sophisticated technologies has shown to be able to deliver the drug in a targeted manner in vitro; and it inhibited tumor growth with therapeutic efficacy and reduced side effects in vivo, showing great promise for the application of nanomedicine in HNC treatment [108].

### 5.2. Enhanced Radiation Therapy

Radiotherapy (RT) is one of the most used treatments in cancer therapy worldwide and remains a significantly effective treatment modality for localized tumors. Over the past few decades, radiotherapy has developed individualized treatments based on anatomical information combined with clinical parameters. Substantial technological advancements that allow real-time imaging and better dose distribution, such as 3D conformal radiation treatments—for example, intensity-modulated radiation therapy (IMRT)—have enabled precise the delivery of high radiation doses to the tumor while reducing the radiation exposure of surrounding healthy tissues [122,123,124]. Due to these advances, the overall survival rates of cancer radiation therapy have improved in the last two decades from about 30% to 80% in HNC [123]. However, challenges such as radioresistance—mainly due to hypoxia, resistant cancer stem cells, and repopulation; and healthy tissue toxicity remain [125]. Owing to this noticeable resistance, nearly 40% of head and neck tumors relapse [125]. 

Recent advances in nanomedicine have allowed the exploration of nanotechnology-based systems aiming to improve radiotherapy through radiosensitization or radioprotection methods. Multiple approaches using nanotechnology have been created to improve radiotherapy as a treatment for HNC; however, in this section, we review only the research that has been conducted using nanoparticles for increased radiosensitization of tumor tissues, taking advantage of the fact that these particles can be ionized and absorb X-rays. Nanoparticles can be composed of elements with high atomic numbers (high-Z nanoparticles). The use of nanoparticles containing elements with high atomic numbers (Z) to enhance the biological efficacy of RT (radiosensitizers) is a promising approach, which may provide effects similar to heavy ion therapy, using a conventional photon beam [126]. In this context, gold nanoparticles have been suggested to improve radiosensitivity [125]. 

Since the 1950s, gold has been recognized as a safe material for biological applications. Its high Z (Z = 79), high capacity for X-ray absorption, versatility, and special physicochemical characteristics allow gold nanoparticles (Au nanoparticles) to have a wide range of practical applications in cancer therapy, namely, as novel radiosensitizers [52,125,127,128]. The radiosensitizing effect of Au nanoparticles is based on the concept that high-atomic-number materials absorb low kilovoltage (kV) X-rays efficiently and deposit plenty of the beam energy in tumor cells, resulting in enhanced radiation dose deposition specifically to tumor cells [129,130]. Since Au nanoparticles are composed of atoms with high atomic numbers (Z), they are very sensitive to radiation, and the photoelectric effect on the material is present. Thus, when irradiated, for each photon incident, a bound electron absorbs its energy and then is ejected from the atom. This effect also happens in other high-Z nanoparticles, being proportional to about Z^4^ [128]. Depending on the type of radiation reaching the metal, secondary particles such as photoelectrons and Auger electrons are produced, and depending on the energy of the beams, reactive oxygen species can be generated, and subsequently, there will be a local dose enhancement [98,99].

In the case of HNC, research into the use of nanoparticles for the improvement of radiation therapies is not yet very advanced. Hainfield et al. evaluated the use of gold nanoparticles for the improvement of radiotherapy (nanogold radiation therapy) in the treatment of highly aggressive and radiation-resistant squamous head and neck carcinoma models (SCCVII). The subcutaneous tumors of SCCVII from twelve rats, after intravenous administration of Au nanoparticles, were irradiated with a superconducting wiggler beam line (42 Gy, 68 keV) for 1 min. The nanoparticles administered had gold cores and were 1.9 nm in diameter (commercially available as AuroVistTM). They used another 12 rats as a control group, and they did not receive the gold injections before the radiation. It was found that in the face of radiation, the volumes of the tumors were successively increased, then decreased, and finally increased; and in the mice that received the nanoparticles the time required for the tumor volume to double was about 76 days, whereas in the mice that did not receive it was about 53 days. However, in the long term (>200 days), 67% of the rats that received the nanoparticles survived, whereas of those that did not received it, only 25% survived [131].

**Figure 6:**
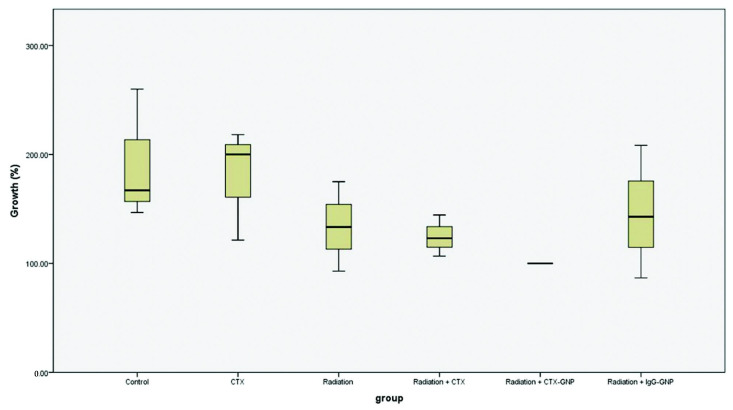
Alterations in tumor growth over 5 weeks of treatment in 6 distinct groups of rats to study ways to overcome tumor radioresistance by increasing absorbed radiation in clinically relevant energy dosages using cetuximab carried by gold nanoparticles. Reprinted with permission from [125].

Therefore, this study showed that the use of Au nanoparticles for RT enhancement for SCCVII is an effective approach. These findings were validated in other studies. Popovtzer et al. (2015) studied how to overcome tumor radioresistance by increasing the absorbed radiation using cetuximab targeted Au nanoparticles. For this purpose, HNSCC cells were injected subcutaneously into 36 mice, and the tumor volume of each was measured. When the tumor reached 8–10 mm, the mice were divided into six groups: group 1 served as the control; group 2 received radiation as a single treatment; group 3, cetuximab (CTX) only; group 4, radiation + CTX; group 5, radiation + IgG-coated (non-targeted) Au nanoparticles; and group 6, radiation + CTX-coated (targeted) Au nanoparticles. In groups 5 and 6, radiation (a single fraction of 25 Gy) was administered 24 h after the injection of Au nanoparticles. Figure 6 shows the changes in tumor growth throughout 5 weeks of treatment. Groups 1–5 showed progression of tumor growth with time. In group 6 (radiation + CTX-Au nanoparticles), the disease remained stable, without presenting any type of progress during the treatment, implying that the CTX-Au nanoparticles were associated with a significant improvement in tumor radiosensitivity relative to other modalities [125]. In the last few years, Au nanoparticles were the most thoroughly studied nanoparticles among those used as radiosensitizers; nevertheless, some studies had shown cytotoxic effects in cells exposed to these nanoparticles [132]. In this context, several alternative high-Z materials began to be studied to avoid those effects, such as gadolinium. Miladi et al. (2015) demonstrated the radiosensitizing effect of gadolinium-based nanoparticles (GBNs in combination with conventional photon radiation in radioresistant HNSCC cell lines). These particles with a diameter of 2.9 ± 0.2 nm were based of a polysiloxane core enclosed by gadolinium chelates covalently grafted to the inorganic matrix. In this sense, the radiosensitizing effect of GBNs on radioresistant cells was studied by evaluating the initial (30 min) and residual (24 h) double-strand DNA breaks (DSB) using the biomarker γ-H2AX foci. In SQ20B cells, 24 h after irradiation, the combination of GBNs with photon irradiation induced a high level of permanent DSBs. This treatment of cells with GBN led to these cells progressing after the G2/M phase after irradiation, and to DNA repair being inhibited, resulting in a mitotic catastrophe, a determining factor for apoptosis [98]. In this sense, Wozny et al. published a work on an experiment they conducted to study the radio-energy effect of these particles. The authors found that GBNs, when combined with the irradiation of carbon ions (^13^C^+6^), is effective for the treatment of HNSCC, leading to increased clinical outcomes of patients [99]. However, gadolinium-based nanoparticles have disadvantages, since gadolinium accumulation in the central nervous system has been described more recently and occurs after repeated administration of this compound. These effects were verified in mice models: prolonged oral administration of gadolinium resulted in locomotor impairment in the exposed animals compared to control animals. These signs thus reflect adverse effects on the central nervous system [133]. In humans it was found that accumulation in the nervous system and consequent nephrogenic systemic syndrome effects occurred in most patients who underwent periodic magnetic resonance imaging (MRI) with gadolinium contrast agents [134,135].

Carbon ion irradiation (one type of hadrontherapy) shows some advantages when compared with conventional radiotherapy because of the particular ballistics (Bragg peak) of carbon ions. In other words, because of focusing energy as much as possible on the tumor site, the surrounding healthy tissues are protected, reducing side effects. Thus, the irradiation of carbon ions has a higher relative biological efficacy (RBE) when compared to conventional RT, since a high local dose is provided along the particle pathways, resulting in complex and irreparable DNA damage and cell death. Wozny et al., in their experiment, used second-generation GBNs containing DOTAGA-(1,4,7,10-tetrakis(carboxymethyl)-1,4,7,10-tetraazacyclododecane glutaric acid)-anhydride (name AGuIX^®^) as a chelator. They estimated the initial DSBs 0.5h after treatment and the residual DSBs 24h after the treatment depending on the amount of γH2AX foci present. Thus, in the first 0.5h, the combination of photons or ^13^C^+6^ radiation with AGuIX^®^ did not significantly increase the number of initial DSBs compared only with irradiation. However, after 24h there was a significant increase in the residual foci in cells treated with AGuIX^®^ plus ^13^C^+6^ and a small increase in AGuIX^®^ plus photons concerning cells irradiated only with ^13^C^+6^ or photons. The enhancement factor for DSBs (EF_DSB_) was calculated by dividing the residual foci after the tested condition for the residual foci after photons. After carbon ion irradiation, the EF_DSB_ value calculated was 1.34 to 2.33 that for the treatment with the addition of AGuIX^®^ to ^13^C^+6^, demonstrating an additive or synergistic effect between AGuIX^®^ and carbon ion irradiation in HNSCC cell lines and better efficiency compared with AGuIX^®^ and photons. Thus, the combination of AGuIX^®^ with photons, and even to a greater extent with ^13^C^+6^ radiation, led to increased residual DSBs, confirming the generation of DNA lesions. The sensitizing effect of AGuIX^®^ is not currently explained, although it is believed that the effect relies on the interaction of the AGuIX^®^ with the electrons produced along the ^13^C^+6^ trajectories [99]. 

Another approach to applying GBNs as a radiosensitizer was performed by Simonet et al., even though the underlying mechanism is not fully known due to the discordant results obtained in vivo. In this study, before the application of radiation, AGuIX^®^ was administered to HNSCC cells. This treatment resulted in their endocytosis and lysosomal accumulation, which alone caused complex DNA damage and induced intracellular ROS production upon radiation exposure, culminating in apoptosis of these cell lines and autophagy. In addition autophagy/autophagic cell death further exacerbated this combination of effects; however, this mechanism is not yet perfectly understood [100].

Hafnium nanoparticles are also currently in clinical trials (phase I/II) for radiation enhancement (NBTXR3) in HNSCC (among others) treatment. NBTXR3 is a radioenhancing hafnium oxide nanoparticle. When sensitized by radiotherapy, it promotes targeted destruction of cancer cells [136,137].

### 5.3. Local Hyperthermia

Hyperthermia (HT) is the exposure of a specific surface or the whole organism to temperatures above the physiological level (between 40 and 45 °C) [138]. The National Cancer Institute distinguishes three types of hyperthermia, considering the location of its application: local hyperthermia, which aims to increase the temperature in a small area; regional hyperthermia, which encompasses larger areas of the body, such as limbs or organs; and total body hyperthermia, generally used for metastatic cancers and as palliative treatment [139]. At the moment, hyperthermia is one of the strategies for the treatment of cancer, alongside surgery, chemotherapy, radiotherapy, and immunotherapy [27]. This therapeutic modality improves clinical responses and reduces the toxicity of conventional treatments. However, from the therapeutic point of view, its use alone as a curative method is not feasible, and it must be combined with chemotherapy and/or radiotherapy [140]. Though this is the only therapy capable of directly attacking the unfavorable tumor microenvironment (hypoxic zones), there is still skepticism regarding the use of hyperthermia [138]. The main limitations in the use of this technique are the lack of precise targeting and optimal temperature control for the target and the surrounding normal tissues. Undirected heating of the tissues can lead to serious damage [27,141]. At relatively high temperatures (above 43 °C), all cells are affected. However, at moderate temperatures (between 39 and 43 °C), normal cells are not sensitive to a temperature rise, whereas tumor cells are. 

The difference targeted by hyperthermia lies in the microenvironments in which the normal and cancerous cells exist. The tumor microenvironment, which presents as hypoxic and acidic, represents a stressor, sensitizing cancer cells to external factors such as high temperatures [142]. The increase in temperature causes a decrease in blood flow in the tumor, which decreases the heat dissipation, leading to a local temperature increase [143]. This increase will provoke the denaturation of the proteins, wjocj results in cellular inactivation through apoptosis (in case of moderate temperatures) and/or necrosis (higher temperatures).

**Figure 7:**
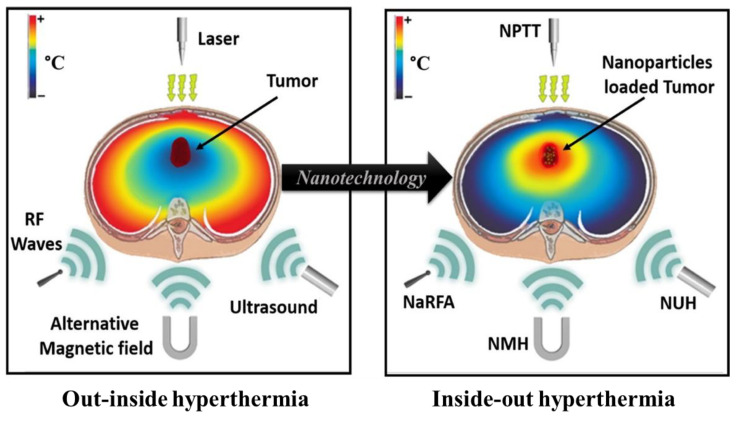
Nanotechnology in local hyperthermia: The nanoparticles are accumulated inside the tumor and are capable of absorbing energy from various external heat sources, thereby potentiating the effects of hyperthermia. On the right, in comparison with the left, it is possible see the effect that the nanoparticles have on heat in the tumor site. Nanoparticles focus the energy from the external source on the tumor to induce localized thermal destruction while minimizing the adverse effects on collateral tissues. Abbreviations: NPTT: nano-photo-thermal therapy. NMH: nano-magnetic hyperthermia. NaRFA: nano-radio-frequency ablation. NUH: nano-ultrasound hyperthermia. Reprinted with permission from [141].

Although the use of hyperthermia for cancer treatment is not new, the challenge of raising the temperature only in target cells is still relevant, and in the past 20 years, several efforts have been made to improve hyperthermia in this way. Advances in nanotechnology have allowed the development of new hyperthermia agents, such as nanoparticles, since these particles can absorb energy from external sources and enhance the effects of hyperthermia. Nanoparticles function as the primary source of heat and reverse the direction of heat loss (Figure 7). Thus, these particles are capable of inducing thermal destruction in a localized manner to the area of interest, thereby minimizing possible effects in adjacent tissues [141]. Local hyperthermia is a great approach for HNC cells, because of their superficial anatomic sites [27].

#### 5.3.1. Plasmonic Photothermal Therapy

Thermal ablation of tumor cells with silica–gold nanoshells (SiAuNS) is a promising approach for the treatment of localized tumors. In plasmonic photothermal therapy (PPTT), the plasmonic nanoparticles, after accumulation in tumors, are irradiated with laser light at specific wavelengths, leading to synchronized oscillation of the conductor band electrons (surface plasma resonance), resulting in light absorption. This absorbed light is transformed into heat, which irreversibly damages the surrounding tissue by the process of necrosis that is induced by heat [27,144]. 

Surface plasma resonance (SPR) is the resonant oscillation of the free electrons present in the conduction band of the nanoparticle atoms induced by the incident light. These electrons absorb the laser photons and get excited at higher energy levels. Through electron–photon relaxation, the absorbed energy of the photon is converted into heat and is transferred to the particle mesh, and consequently to the tumor tissue. Thus, the interactions of these electrons with the incident light result in several thermal, optical, and electrical properties [144]. The most extensively studied nanoparticles for this purpose are Au nanoparticles, due to their biocompatibility. Additionally, by changing the morphology (size, composition, shape) of the nanoparticles, the wavelength of light with which the nanoparticle absorbs maximal energy (SPR peak) can be adjusted to visible or near-infrared (NIR) (650–900 nm), by which the penetration of tissues by light is maximal [145]. Thus, PPTT using NIR is a promising approach for HNC, as it can improve many treatments, through the accumulation of PPTT agents at the target site and localized laser irradiation, minimizing thermal damage to healthy tissues. 

Trinidad et al. developed silica–gold nanoshells (SiAuNS) each composed of a dielectric core (silica) coated with a gold layer capable of absorbing light with wavelengths located in the NIR region for PPTT. In their experiment, they used SiAuNS-loaded macrophages (Ma) for nanoparticle delivery, as Ma are attracted to hypoxic and necrotic regions in tumors. They examined the effects of SiAuNS-loaded Ma mediated PPTT and photodynamic therapy (PDT), separately and in combination on HNSCC cells. Both treatments rely on irradiation of the tumor region with light in the NIR region, which activates photoresponsive molecules (in this case, AlPcS2a), which induce the production of ROS (PDT) or a temperature increase (PPTT). Two different wavelengths of light were used concurrently, 670 nm to activate the PDT photosensitizer, and 810 nm for the SiAuNS-loaded Ma (PPTT), to evaluate the combined effects of these techniques. Initially, the uptake of SiAuNS by the macrophages was assessed, which endorsed the possibility of their use as efficient transport vectors. The sustainability of the SiAuNS-loaded Ma was reduced to 35% or 12% of the control values with irradiance (5 min) producing heat flux density of 14 or 28 W/cm^2^, respectively; but no significant cytotoxicity was detected for non-loaded Ma under the same conditions of PPTT exposure. Therefore, the uptake of SiAuNs by the macrophages was verified. To check the effectiveness of these treatments in HNSCC, FaDu and SiAuNS-loaded Ma were combined at two different ratios, 1:1 and 2:1 (FaDu:MaNS). At a 1:1 ratio and with 14 W/cm^2^ irradiance, 50% of the cells survived, compared to only 35% for the AuNS-loaded PPTT treated cells. At an irradiance level of 28 W/cm^2^, there was no alteration difference in the PTT effects on AuNS-loaded Ma and the combined cells [101].

The combination of PPTT and PDT was studied, with a 2:1 FaDu:MaNS cells ratio. A substantial decrease in cell viability was reached at 14 W/cm^2^, with 810 nm laser irradiance and a PDT radiant exposure of 0.25 J/cm^2^. At lower PTT irradiances, no significant cytotoxic effects of PTT plus PDT were detected. The degree of synergism for combined PTT-PDT treatments was calculated and compared with single treatments. If the result was greater than 1, it was synergistic, less than 1, antagonistic, and equal to 1, additive. The ratio values for the effects of combined treatment cytotoxicity for PPTT irradiance of 14 W/cm^2^ ranged from 1.83 ± 0.16 for the cells that received PDT radiant exposures of 0.5 J/cm^2^ to 2.29 ± 0.26 for the cells that received 0.25 J/cm^2^, indicating a clear synergistic effect [101].

**Figure 8:**
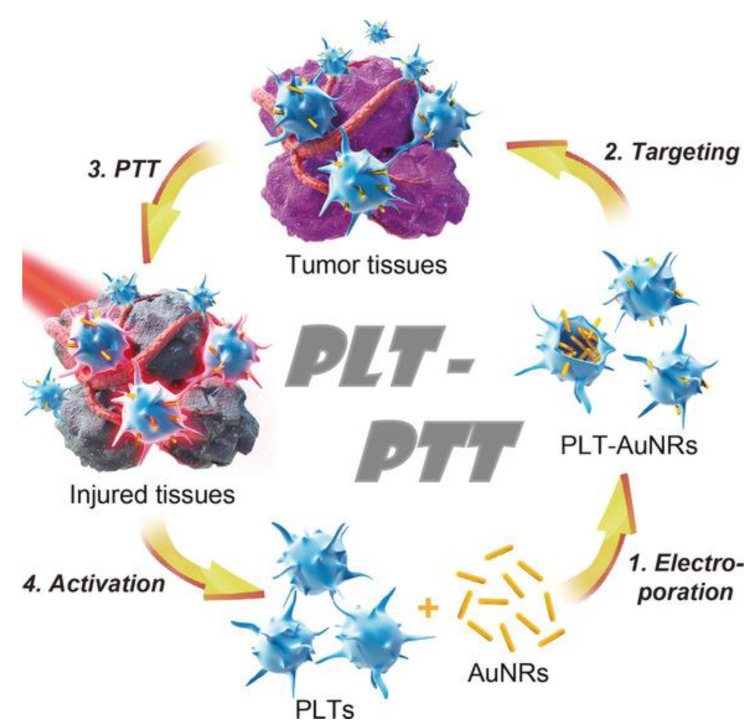
Platelet-facilitated photothermal tumor therapy (PLT-PTT): Platelets (PLTs) after isolation in the blood were mixed with gold nanorods (AuNRs), which after an electroporation process, were taken up by the PLTs. The resulting AuNR-loaded PLTs (PLT-AuNRs) reached the tumor cells using in vivo photothermal tumor therapy (PTT). Reprinted with permission from [102].

Gold nanorods (AuNRs) are of great interest for PPTT, as their SPR peak is in the NIR region. However, without a strategy to facilitate their targeting to tumor tissues, the AuNRs are dependent on the EPR effect to reach the target. Additionally, like many other external biomaterials, AuNRs can be easily recognized by the immune system. Thus, similarly to Trinidad et al., Rao et al. used a platelet-facilitated photothermal tumor therapy (PLT-PTT) strategy where platelets were carriers of Au nanoparticles to the tumor tissue. Platelets are circulating sentinels that accumulate in ill tissues and induce the repair processes required by injured tissues. In addition, PTT-mediated heat is thought to injure tumor tissue, so PLTs are even better for AuNRs accumulation. Therefore, AuNRs were loaded into PLTs (Figure 8) through the electroporation process, resulting in AuNR-loaded PLTs, with long-circulating and cancer targeting capabilities, and were used in PPTT (submitted to NIR irradiation) [102].

The photothermal conversion was evaluated after irradiation with an NIR laser (808 nm). As expected, the temperature of the phosphate-buffered saline (PBS) containing AuNRs increased 35 °C, with AuNR-loaded PLTs presenting equivalent results, thereby showing that both can efficiently convert energy into heat. AuNRs also showed some biocompatibility that was enhanced when loaded into PLTs. Likewise, the cell uptake was studied by incubating the nanoparticles with macrophage-like cells. AuNRs showed the highest uptake rates. AuNR-loaded PLTs showed lower uptake, demonstrating their capacity to avoid phagocytosis. Biodistribution studies showed that AuNR-loaded PLTs exhibited systematic circulation after 48 h, suggesting evasion capacity. Additionally, it was found that PPTT treatment increased the targeting of AuNR-loaded PLTs to the tumor, because of PLTs attraction to injured sites, indicating the unique self-reinforcing characteristic of PLT-PPTT in cancer therapy. The administration of AuNR-loaded PLTs and localized laser irradiation effectively inhibited the growth of HNSCC cells [102].

The efficiency of Au nanoparticles as photothermal agents when irradiated with a laser with a wavelength of 530 nm in oral squamous carcinoma cells (OSCC) that overexpressed EGFR was assessed by El Sayed et al. [103]. For this, Au nanoparticles were conjugated with anti-EGRF antibodies to improve specificity for cancer cells due to the overexpression of EGFR on the surface of the cancer cells. After successful recognition of OSCC cells, they were exposed to a laser (various power densities), to evaluate the effect of Au nanoparticles on cell viability. As expected, the malignant cells suffered irreversible photothermal injury at a much lower power density than the non-carcinoma cell lines due to the greater affinity of the nanoparticles towards the EGFR present on the surface of cancer cells [103]. 

In another study, based on Rose Bengal (RB) specificity toward oral cancer, Wang et al. (2013) employed RB-conjugated AuNRs in photodynamic and photothermal oral cancer therapies [104]. Under 532 nm irradiation, RB-conjugated AuNRs exhibited efficient singlet oxygen generation, which proves their value as photodynamic agents. Additionally, under 810 nm light irradiation, their temperature increased by 43.3 °C, whereas that of water only increased by 2.2 °C, indicating the efficient conversion of NIR light into thermal energy, demonstrating their value as photothermal agents. Significant anticancer effects were observed from the RB-conjugated AuNRs during combined photothermal and photodynamic therapy, confirmed in vitro and in vivo [104].

#### 5.3.2. Magnetic Fluid Hyperthermia

The use of biocompatible magnetic nanoparticles for cancer treatment to induce localized heating has been the subject of much attention by the research community. The first application of magnetic materials as hyperthermia agents dates to 1957 when Gilchrist et al. used magnetite particles to increase the temperature of several tissue samples when exposed to a magnetic field [146]. Meanwhile, several studies have been published that describe a variety of experiments using different types of magnetic materials, such as various types of nanoparticles [143,147], together with radiotherapy or chemotherapy [148,149], to check the effect of hyperthermia [150].

Superparamagnetic nanoparticles are each composed of a magnetic core-shell and a polymer coating that will hold various therapeutic and targeting moieties. They are selectively targeted to a tumor site either passively, using their inherent structural properties (passive targeting), or actively by using other means. These systems have intrinsic magnetic properties that allow them to be used as contrast agents in MRI alongside with hyperthermia, and their biocompatibility allows them to act as drug carriers in a targeted manner by using an external magnetic field centered on the lesion [151]. Magnetite (Fe_3_O_4_) and maghemite (γ-Fe_2_O_3_) are the materials most widely used in superparamagnetic nanoparticles for biomedical applications [152]. When subjected to an alternating magnetic field (AMF), they dissipate thermal energy (produce heat) by three loss mechanisms, depending on the size of the particles and their state of domain: losses of hysteresis in bulk multi-domain magnetic materials, generation of heat due to eddy currents, or relaxation losses in a single-domain superparamagnetic particles (below 100 nm) [153].

Nanoparticles with superparamagnetic behavior only present magnetization in the presence of an external field [153]. The fact that they do not present permanent magnetism adds value, since it prevents their aggregation [154,155]. One of the benefits of magnetic nanoparticles is that they release heat as a result of loss of hysteresis, Neel relaxation, and Brownian relaxation when under the influence of an alternating magnetic field [156,157]. The Neel relaxation is due to the reorientation of spin according to the AMF after each oscillation; the Brownian relaxation refers to the friction between the particle and the medium into which it is inserted. The Neel relaxation and Brownian relaxation are dependent on particle size, and the latter is also dependent on the viscosity of the medium [153]. For smaller particles, the mechanism of relaxation of Neel prevails, being, therefore, the main one responsible for the release of heat by the magnetic nanoparticles [143]. This type of nanoparticle can release heat when an AMF is applied, delivering the adequate thermal dosage to the target site, while sparing healthy tissues through an inside-out hyperthermia approach called magnetic fluid hyperthermia (MFH) [153]. MFH has been approved in Europe for the treatment of multiform glioblastoma in a methodology that requires direct delivery of the nanoparticles to the tumor tissue [158].

In HNSCC, cancer stem cells (CSCs) having, for example, the cell-surface glycoprotein CD44, have an integral role in tumor progression, metastasis, and treatment resistance. Thompson et al. explored new ways to target the CD44 population for both treatment and imaging by using dextran-coated superparamagnetic iron oxide nanoparticles functionalized with hyaluronic acid (HA)-HA-DESPIONs. Tongue squamous cell carcinoma cells (UT-SCC-14) were used to evaluate the hyperthermic capacity of HA-DESPIONs or non-HA-coated DESPIONs, when exposed to an AMF. Dual staining of CD44 and apoptosis showed that cell death was increased only when the magnetic field was activated and in the CD44 positive population that was exposed to HA-DESPIONs. The apoptotic rate increased from 1.4% to 27.5% in the CD44 positive cells, indicating the successful targeting of HA-DESPIONs to UT-SCC-14 cells, due to the overexpressed surface receptor CD44. The activation of HA-DESPIONs due to the presence of an AMF generated a significant localized temperature increase that ultimately led to tumor cell death [105]. Another work that showed advances in fighting and killing CSCs resistant to conventional therapies was that performed by Su et al. (2019), in which they also targeted the CD44 population by using MFH. They developed a method to prepare anti-CD44 antibody-modified superparamagnetic iron oxide nanoparticles that are able, through the application of an MFA, to penetrate CSCs, causing their apoptosis, and give rise to necrotic areas distributed around the magnetic fluid in tumor tissue. This strategy was shown to inhibit the growth of grafted Cal-27 tumors in mice while at the same time proving to be biocompatible [106]. This validates how the identification of these markers in CSCs plays a key role in the efficacy of killing CSCs that accumulate in tumor tissues and are resistant to conventional therapies.

In another study, Zhao et al. evaluated the use of magnetic iron oxide nanoparticles induced hyperthermia for treatment of HNC using a mouse xenograft model of an HNSCC cell line (Tu212). Nanoparticles were delivered intratumorally to protect other tissues and improve the hyperthermic effect. According to histopathology results, there was ulceration in the walls of the treated tumors (not seen in the nontreated controls). Additionally, the presence of necrosis of the epithelium in the wall of one treated tumor indicates that hyperthermia-mediated cell death is due to necrosis [107]. These results show promising strategies for using targeted magnetic nanoparticles to combat tumor progression, metastasis, and treatment resistance.

### 5.4. Theragnostics

Nanoparticles are used not only for therapeutic and imaging purposes, but also theragnostic approaches, that is, for approaches that combine diagnosis and therapy in one single system. Theragnostic platforms, such as liposome, gold, and iron-based nanoparticles, among others, can provide passive and active targeting, trigger drug release, and perform other therapeutic functions, and at the same time are less invasive than conventional diagnostic systems [159,160].

**Figure 9:**
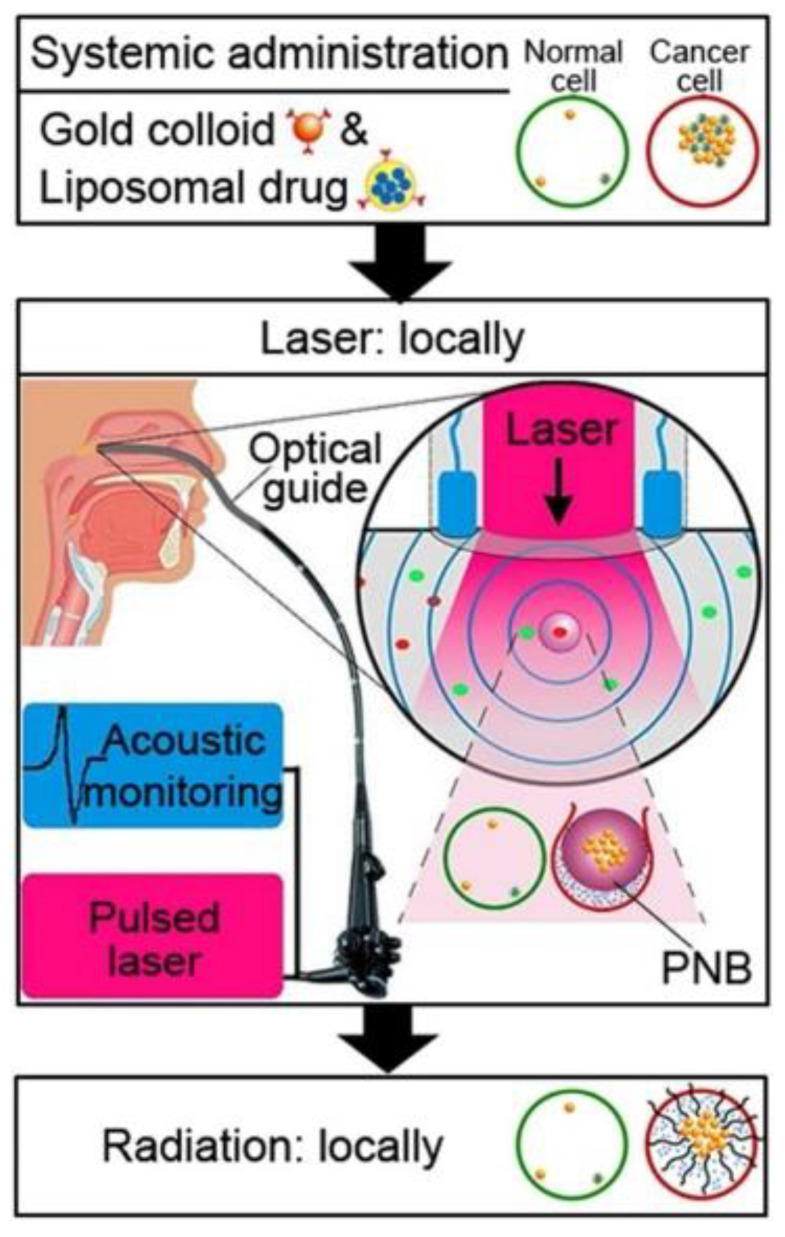
Quadrapeutic strategy in cancer treatment: Systemic administration of gold colloids conjugated with antibodies and liposomal drugs to form nanoclusters in cancer cells; local application of a laser pulse through an endoscope to selectively generate plasmonic nanobubbles (PNBs) in cancer cells; selective amplification of radiation by the nanocluster in cancer cells. Reprinted with permission from [161].

In HNC therapy, there are some multifunctional nanocarriers for theragnostic purposes, such as quadrapeutics, which can be applied in the detection and eradication of HNSCC [161]. Quadrapeutics are combinations of four therapeutic approaches: drug delivery, Au nanoparticles, NIR laser, and radiation therapy. This therapeutic strategy is based on three stages (Figure 9): aggregation of a nanocluster of Au nanoparticles and a liposomal drug (both conjugated to an antibody specific to that cancer); its endocytosis mediated by receptors on tumor cells, with some normal cells being affected but resulting in smaller nanoclusters; application of the NIR laser—its energy is converted into heat due to the SPR effect which promotes the formation of plasmonic nanobubble (PNB), which are steam nanobolines produced transitorily around super-heated plasmonic nanoparticles that act as mechanical therapeutic agents capable of destroying the cell and releasing the liposomal drug. This nanocluster selectively amplifies the radiation. This combination of steps inside the cell of the liposomal drug gold, laser, and radiation has a synergistic effect, resulting in significant therapeutic effects on cancer cells. According to in vivo tests of a primary and microscopic residual HNSCC, this therapeutic strategy has been shown to increase the effectiveness of standard chemotherapy by more than 17 times while using only about 3% to 6% of the normal clinical doses of chemotherapy and radiation, without causing side effects or residual tumors [161].

Muhanna et al. (2015) assessed (1) the effectiveness of porphysome nanoparticles for enhancing fluorescence and photoacoustic imaging of head and neck tumors and (2) the effectiveness of this agent for localized photothermal ablative therapy. The results show that porphysomes enabled imaging of head and neck carcinomas and the targeted ablation of these tumors [162]. Another approach was the work performed by Davidi et al., in which they developed a unique nanoplatform consisting of gold nanoparticles coated with cisplatin and glucose, which works simultaneously as a radiosensitizer and as a drug carrier that specifically delivers cisplatin to the tA431 HNSCC tumor cells. This nanoplatform also showed an efficient ability to detect tumor imaging. Therefore, it has a synergistic therapeutic effect, since it works not only as a vehicle of the therapeutic agent but also as an efficient contrast agent for the tumor images. It showed efficient penetration into tumor cells and cellular toxicity equivalent to that of cisplatin alone. Furthermore, when combined with radiation, it resulted in greater tumor reduction than either radiation and free cisplatin or radiation alone. Thus, this work showed that this approach using a single nanoparticle formulation has the potential to enhance the antitumor effect while overcoming resistance to chemotherapeutics and radiation, and also enables better diagnosis and image-guided therapy, making it a promising theragnostic agent [163].

Li et al. studied a non-invasive technology-targeted strategy to overcome some existing drawbacks of OSCC diagnosis, for which so far a pathological biopsy is still the current gold standard. Taking advantage of the fact that peptide-releasing gastrin receptor (GRPR) is overexpressed in HNSCC and acts as a potential target for OSCC fluorescent optical imaging, this research team produced non-graphene oxide nanoparticles (NGOs) coupled with AF750-6Ahx-Sta-BBN, which resulted in NGO-BBN-AF750, a probe with immunofluorescence capabilities. They studied their GRPR binding, uptake, and internalization in HSC-3 cells for early OSCC diagnosis, and the results showed that NGO-BBN-AF750 and AF750-6Ahx-Sta-BBN have similar binding affinity to GRPR in HSC-3 cells. On the other hand, NGO-BBN-AF750 showed good cellular internalization capabilities compared to the peptide antagonist AF750-6Ahx-Sta-BBN. This work revealed how nanoparticle-based delivery systems can enable OSCC-targeted near-infrared fluorescence imaging in a non-invasive manner [164].

Lastly, magnetic nanoparticles are promising nanotechnology-based systems, used both in therapy (through drug delivery and/or magnetic fluid hyperthermia) and in magnetic particle imaging (MPI). The combination of magnetic nanoparticles’ unique properties for theragnostic purposes was studied by Hensley et al., as the physics behind both processes are similar. The magnetic field used in MPI can also be used in MFH; thus, they investigated the use of MPI systems in therapeutic MFH, reporting the first combined MPI–MFH system, which demonstrated the selective heating of target cells. Thus, in this work, a new diagnostic imaging modality, MPI, was introduced that enables a focused theragnostic approach encompassing treatment planning, treatment control, and spatially localized inductive heating. This novel nanoparticle design facilitates efficient targeting of the nanoparticles to the tumor required in MFH, and other therapeutic combinations may give rise to safer alternatives for cancer treatment [165]. 

## 6. Challenges and Opportunities in the Application of Nanocarriers in HNC

In contrast to the conventional therapeutic approaches to HNC treatment, nanomedicine-based therapeutics have faced several challenges during clinical development. Several needs and opportunities have been identified that may be beneficial to improving their efficacy and safety for cancer treatment: greater precision in characterizing the sizes, shapes, and compositions of these systems; development of reproducible and scalable production of nanomedicines with optimized bioavailability and excretion profiles at the industrial level; reductions in the polydispersity of colloidal systems and improvements in the stability of formulations; understanding of the complexity of this type of cancer and importance of the structures involved for vital functions; designs for systems that cross biological barriers and induce only low levels of cytotoxicity in adjacent, healthy head and neck tissues; understanding of the exact interactions of nanomedicines with their biological environments; optimization of selectivity for maximum targeted delivery through surface ligands, avoiding non-specific adsorption in other tissues; methodology for the quantification of the ligand molecules and their distribution on the surfaces of these nanocarriers; development of nanomedicines with multivalent properties and specificity for various receptors as a treatment strategy for several therapies through a single formulation [9,166,167,168,169,170]. 

Concerning the pharmacokinetics, biodistribution, and residence times of nanocarriers in the body, it was found that their biopersistence is high and that sometimes problems arise in their clearance, making these aspects also reliant on the design and development of such systems [167,171]. One of the factors with a significant impact on the excretion of these nanomaterials is particle size. In this respect, it was found that in general, the hydrodynamic diameter set as the maximum for excretion via the kidneys (renal clearance) is 10 nm. Colloidal species with larger sizes tend to be filtered via the endothelial reticulum system or via the liver. This can be illustrated by MRI contrast agents based on dendrimers of PAMAM. As a function of their size, keeping all other parameters equivalent, it was found that dendrimers with less of 10 nm dendrimers were eliminated via the renal route, whereas larger dendrimers were eliminated via the liver [167,172]. This is important because the accumulation of nanomedicines in healthy cells and organs can result in cytotoxicity, which could result in a variety of adverse effects. One relevant examples is the bioaccumulation of colloidal Au. Not all circulating Au nanoparticles are eliminated by hepatobiliary excretion, so many end up accumulating in the spleen, liver, and mesenteric lymph nodes, in which their metabolization or excretion as ionic Au can take many months. On the other hand, Au nanoparticles internalized in cells are also not easily eliminated, although there is some evidence for size-dependent exocytosis [167,173,174].

Other factors affecting the extension of clearance are nanoparticle structure and material type. Staal et al. showed that multi-core PLGA nanoparticles containing perfluoro-15-crown-5 ether (PFCE) have a 15-fold reduction in vivo half-life compared to what is described for other systems of this diagnostic agent. Thus, they found accelerated hepatic clearance due to the disassembly of the ~200 nm nanoparticle into ~30 nm domains that make it more soluble, facilitating elimination. Thus, the ultrastructure of the nanoparticle has a direct impact on the in vivo release of its contents [175].

To circumvent all these problems, Dong et al. produced thermo-organic inorganic ultrasound nanosystems for multiple bioimage-guided cancer nanotherapeutics that were biodegradable and easily excretable. Thus, this group proposed a multifunctional theranostic nanosystem based on degradable and excretable ultrasmall transition metal selenide nanodots with a general bovine serum albumin-templated strategy to be used in high performance computed tomography [176]. 

Torresan et al. also showed in in vivo tests how nonequilibrium Au-Fe nanoalloys behave as 4D multifunctional models, since they can change shape, size, and structure over time. They can transform into nanocrystals when used as contrast agents for magnetic resonance imaging and computerized X-ray absorption for photography. These systems over time have been shown to have self-degrading capabilities. Their accumulation in the body reduces faster over the long term when compared to other gold or iron oxide contrast agents [177].

Therapeutics researchers will have several opportunities to improve personalized medicine, once we can develop systems not just for diseases, but ones which are patient-specific, since some systems could target specific markers. Given the impact of HNC and the emergence of treatments providing better therapeutic outcomes, it is also important that researchers and the pharmaceutical industry study nanoparticle screening and evaluation more, and that we see improvement in governmental support for the development of these promising nanoformulations. We would also like to see governments backing their scaled-up production, which will also their approval beforehand [48,166].

Another focus in this in this field is developing new methods or tools to access and monitor the behavior that nanocarriers have in the human body (biodistribution patterns) during their transport to tumors. Additionally, it is important to know their interactions with organ and biomarker systems in vivo [168].

## 7. Conclusions

Head and neck cancer is one of the most common cancer types worldwide, and its incidence is expected to increase in the future. Despite the latest advances in medicine, cancer survival rates have remained unaltered for the past 25 years, mostly due to the lack of efficiency in drug targeting, which leads to ineffective therapy. Due to the problems associated with HNC treatment (comorbidities, compromised organ function, etc.), there is a tremendous need for innovative approaches in cancer management, due to difficulties in drug targeting. Currently, only low concentrations can be produced in the target zone, compromising therapeutic efficacy. Advances have been made that not only increase the concentration of a drug in the target site, but also provide personalized therapy. In this context, nanomedicine via nanotechnologies emerged as a powerful ally to achieve these goals through the development of nanocarriers that can go unnoticed to the immune system and are also led to a tumor by various targeting strategies. Additionally, they have the potential to improve the efficiency of chemotherapy without increasing the toxicity to healthy tissues. In addition, these nanocarriers allow simultaneous delivery of anticancer drugs, which makes it easier to combat tumor resistance. Besides treatment, nanotechnology can provide us the tools to shift the paradigm of HNC management through theragnostic strategies, which allow simultaneous diagnosis and therapy. However, there is further work needed to understand the toxicity, biocompatibility, long-term implications, and limitations of the nanocarriers proposed so far.

## Figures and Tables

**Table 1:** Tumor classification according to TNM profile [39,43]. T—extent of the primary tumor, N—infected regional lymph nodes, M—distant metastases.

Stage	T	N	M
0	Defined shape	No invasion	No distant metastasis
I	Defined shape, less than 2 cm Does not invade the submucosa	No invasion	No distant metastasis
II	Between 2 and 4 cm Initial invasion of the submucosa	No invasion	No distant metastasis
III	Cancer cells rapidly divide Tumors with more than 4 cm	Invasion	No distant metastasis
IV	Cancer cells enter the bloodstream	Invasion	Distant metastasis

## Data Availability

Not applicable.

## Abbreviations and Acronyms

AuNRs—Gold nanorods; AuNS—Gold nanoshells; BLM—Bleomycin; CDF—Curcumin-difluorinated; CSCs—Cancer stem cells; CTX—Cetuximab; DDP—Cisplatin; DDAB—Dimethyldioctadecyl ammonium bromide; DOTAGA—(1,4,7,10-tetrakis(carboxymethyl)-1,4,7,10-tetraazacyclododecane glutaric acid)–anhydride; DSBs—Double-strand DNA breaks; EBV—Epstein-Barr virus; EGFR—Epidermal growth factor receptor; EMT—Epithelial–mesenchymal transition; EPR- Enhanced permeability and retention effect; FDA—Food and Drug Admnistration; GBNs—Gadolinium-based nanoparticles; HNC- Head and neck cancer; HNSCC—Head and neck squamous cell carcinoma; HPV—Human Papillomavirus; HT—Hyperthermia; ICIs—Immune checkpoint inhibitors; IMRT—Intensity-modulated radiation therapy; MPI—Magnetic particle imaging; MTX—Methotrexate; NIR—Near infrared radiation; NLC—Nanostructured lipid carriers; PAA—Polyacrylic acid; PAMAM—Polyamidoamine; PBS—Phosphate-buffered saline; PDT—Photodynamic therapy; PD-L1—Programmed death-ligand 1; PEG—Polyethylene glycol; PLGA—Poly(lactic-co-glycolic acid); PLT—Platelets; PNBs—Plasmonic nanobubbles; PPTT—Plasmonic photothermal therapy; Pt—Platinum; PTTC—Photothermal tumor therapy; PTX—Paclitaxel; QDs—Quantum dots; RBE—Relative biological efficacy; RT—Radiotherapy; SAS—Human tongue squamous cancer; SiAuNS—Silica-gold nanoshells; SPR—Surface plasma resonance; TNM—Tumor, node, metastasis; TME—Tumor microenvironment factors; VEGF—Vascular endothelial growth factor; α-TOS—α-tocopheryl succinate.
